# Refuting the challenges of the developmental shift of polarity of GABA actions: GABA more exciting than ever!

**DOI:** 10.3389/fncel.2012.00035

**Published:** 2012-08-28

**Authors:** Yehezkel Ben-Ari, Melanie A. Woodin, Evelyne Sernagor, Laura Cancedda, Laurent Vinay, Claudio Rivera, Pascal Legendre, Heiko J. Luhmann, Angelique Bordey, Peter Wenner, Atsuo Fukuda, Anthony N. van den Pol, Jean-Luc Gaiarsa, Enrico Cherubini

**Affiliations:** ^1^INSERM Unité 901, Université de la Méditerranée, UMR S901 Aix-Marseille 2 and INMEDMarseille, France; ^2^Department of Cell and Systems Biology, University of TorontoToronto, ON, Canada; ^3^Institute of Neuroscience, Medical School, Newcastle UniversityNewcastle upon Tyne, UK; ^4^Department of Neuroscience and Brain Technologies, The Italian Institute of TechnologyGenova, Italy; ^5^Centre National de la Recherche Scientifique, Institut de Neurosciences de la Timone, UMR 7289, Aix Marseille UniversitéMarseille, France; ^6^INSERM U 952, Centre National de la Recherche Scientifique, UMR 7224, Université Pierre et Marie CurieParis, France; ^7^Institute of Physiology and Pathophysiology, University Medical Center of the Johannes Gutenberg-University of MainzMainz, Germany; ^8^Department of Neurosurgery and Cellular and Molecular Physiology, Yale University School of MedicineNew Haven, CT, USA; ^9^Department of Physiology, Emory University School of MedicineAtlanta, GA, USA; ^10^Department of Neurophysiology, Hamamatsu University School of MedicineHamamatsu, Japan; ^11^Department of Neurosurgery, Yale University School of MedicineNew Haven, CT, USA; ^12^Department of Neurobiology and Italian Institute of Technology Unit, International School for Advanced StudiesTrieste, Italy

**Keywords:** GABA, giant depolarizing potentials, energy substrates, brain slices, chloride homeostasis, development

## Abstract

During brain development, there is a progressive reduction of intracellular chloride associated with a shift in GABA polarity: GABA depolarizes and occasionally excites immature neurons, subsequently hyperpolarizing them at later stages of development. This sequence, which has been observed in a wide range of animal species, brain structures and preparations, is thought to play an important role in activity-dependent formation and modulation of functional circuits. This sequence has also been considerably reinforced recently with new data pointing to an evolutionary preserved rule. In a recent “Hypothesis and Theory Article,” the excitatory action of GABA in early brain development is suggested to be “an experimental artefact” (Bregestovski and Bernard, 2012). The authors suggest that the excitatory action of GABA is due to an inadequate/insufficient energy supply in glucose-perfused slices and/or to the damage produced by the slicing procedure. However, these observations have been repeatedly contradicted by many groups and are inconsistent with a large body of evidence including the fact that the developmental shift is neither restricted to slices nor to rodents. We summarize the overwhelming evidence in support of both excitatory GABA during development, and the implications this has in developmental neurobiology.

During brain development there is a progressive shift in the pattern of network activity toward an adult form that is sustained by a sequence of gradual changes in voltage- and transmitter-gated currents. Immature currents are sluggish with a long time course that enables very heterogeneous neurons to fire and connect together. To the best of our knowledge, there is not a single current or pattern that is identical in immature and adult networks: an immature brain is clearly not a small adult brain. One of the best-documented developmental sequences in physiology is the progressive reduction of intracellular chloride in neurons and the associated switch in GABA polarity (Obata et al., 1978; Mueller et al., 1984; Ben-Ari et al., 1989, 2007; Owens et al., 1996; Ben-Ari, 2002; Owens and Kriegstein, 2002; Tyzio et al., 2008; Blaesse et al., 2009). This has been confirmed in a wide range of animal species from worms to higher mammals, brain structures and preparations from neuronal cultures to slices, intact organs *in vitro* and *in vivo* [see the large table with the papers showing the developmental sequence in Ben-Ari et al Physiological reviews (Ben-Ari et al., 2007)]. The activation of GABA_A_ and glycine receptors during early postnatal development routinely produces membrane depolarization, which, in some occasion, reach spike threshold to generate sodium action potentials (Chen et al., 1996; Khazipov et al., 1997; Leinekugel et al., 1997; Mienville, 1998; Dzhala and Staley, 2003), the activation of the non-inactivating sodium currents (Valeeva et al., 2010), the activation of voltage gated calcium currents (Leinekugel et al., 1997) and the removal of the voltage dependent Mg^++^ block of NMDA channels also leading to large calcium influx (McLean et al., 1996; Leinekugel et al., 1997; Caillard et al., 1999). The GABA/NMDA links (Ben-Ari et al., 1997) has been reinforced recently with immuno-cytochemical observations (Cserep et al., 2012). Depolarizing GABA during development and the subsequent shift to inhibitory transmission are widely accepted as key events in the proper development of neuronal networks and brain structures (Ben-Ari, 2002; Owens and Kriegstein, 2002). Brain development is also associated with the generation by depolarizing GABA of immature network patterns like the Giant Depolarizing Potentials (GDPs) in the hippocampus (Ben-Ari et al., 1989) and other brain structures (Ben-Ari, 2001, 2002). GDPs also parallel long lasting brain patterns that are present in the developing but not the adult brain (Ben-Ari, 2001, 2002).

During the last two decades, this sequence of events has been strongly reinforced by several complementary observations including: (1) the developmental sequence of the chloride co-transporters NKCC1 and KCC2 expression that has provided a mechanistic substrate to the progressive reduction of intracellular chloride (Rivera et al., 1999; Payne et al., 2003; Yamada et al., 2004; Blaesse et al., 2009); (2) the demonstration that GABAergic currents mature before glutamatergic ones providing the first and sole source of activity in various brain structures (Chen et al., 1996; van den Pol et al., 1998; Tyzio et al., 1999; Gao and van den Pol, 2001; Hennou et al., 2002; Gozlan and Ben-Ari, 2003; Johnson et al., 2003; Wang and Kriegstein, 2008); (3) the demonstration that GABAergic hub interneurons orchestrate the generation of GDPs that represent the first synapse-driven patterns of activity in the hippocampus (Bonifazi et al., 2009; Picardo et al., 2011); (4) the findings that early in development, before the establishment of synapses, growth cones contain and release GABA and respond to GABA with calcium elevations (Obrietan and van den Pol, 1996; van den Pol, 1997; Gao and van den Pol, 2000) and; (5) the demonstration of an oxytocin mediated abrupt shift during delivery that exerts a neuroprotective and analgesic action on the newborn's brain (Tyzio et al., 2006). Collectively, these observations have provided a general concept for the development of cellular and network activities and how they modulate the construction of neuronal ensembles. The GABA developmental shift of polarity has been accepted by a wide range of researchers and considered as a fundamental property of developing networks (Ben-Ari et al., 2007).

In a recent review article, Bernard and Bregestovski challenge this ensemble of observations claiming that excitatory GABA is a “correct observation but an experimental artifact” (Bregestovski and Bernard, 2012). This paper relies on two sets of observations:

1. The observations made by Zilberter et al. that the depolarizing/excitatory actions of GABA in glucose perfused slices shift to hyperpolarizing/inhibitory in the presence of additional Energy Substrates (ESs) including ketone bodies metabolites lactate or pyruvate (Holmgren et al., 2010; Zilberter et al., 2010; Mukhtarov et al., 2011). The authors suggest that neurons are energy deficient in glucose-perfused slices resulting in the depolarizing action of GABA. ESs are also suggested to better mimic maternal milk than glucose and therefore to be physiologically relevant.
2. The observations by Staley et al. (Dzhala et al., 2012) that the slicing procedure damages surface, but not deep, neurons in neoatal slices leading to an accumulation of intracellular Cl^−^. Bregestovski and Bernard (2012) extend this observation to their model suggesting that injured (surface) neurons have high-energy requirement that are not met by glucose, resulting in the excitatory actions of GABA and the associated generation of GDPs.

Challenging an established concept is always good news in science as it stimulates discussion and the elaboration of novel concepts. However, this review and the underlying observations fall short of challenging the developmental sequence of the polarity of GABA actions as they are based on a small set of studies that have been repeatedly invalidated by many groups. This review has also overlooked extensive investigations that directly contradict the studies that are cited in addition to important contradictions. We shall first examine the validity of the ESs results and the damage explanation, before analysing more general aspects of the proposed model.

## The role of energy substrates in GABA polarity

**Figure 1:**
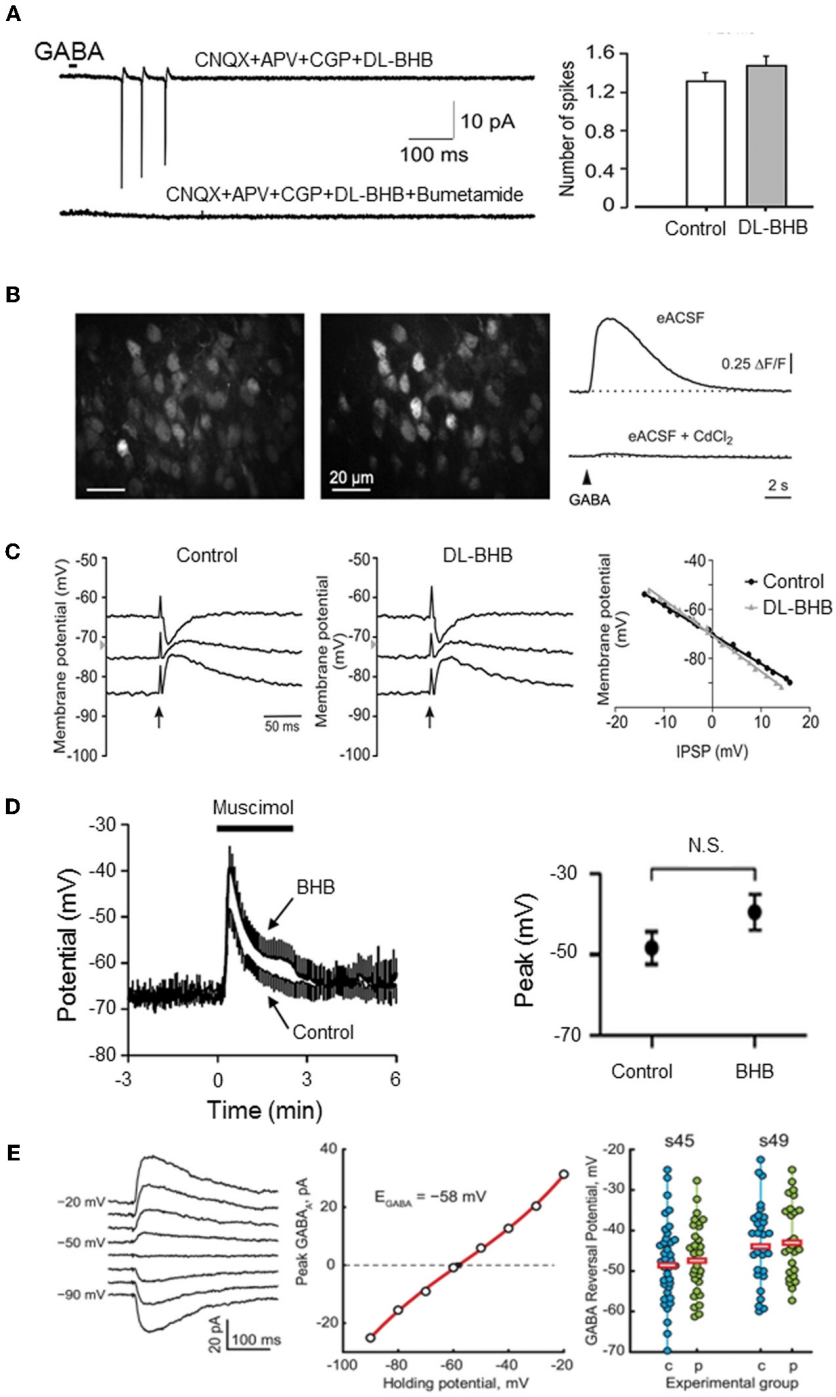
**Energy substrates do not affect E_GABA_ or GABA actions in the immature neurons. (A)** Energy substrates (DL-BHB, 4 mM) do not alter the excitation produced by focal application of GABA in the neonatal rat hippocampus (cell-attached patch-clamp recording). Bumetanide (10 μm) fully blocked the GABA response. [Modified from Tyzio et al. (2011)]. **(B)** Energy substrates (DL-BHB, 4 mM) do not affect GABA-mediated Ca^2+^ in neonatal cortical mice. Raw fluorescence image displaying OGB1-stained cells in the upper cortical plate (left) and ΔF image illustrating GABA-responsive cells (middle). GABA-mediated somatic [Ca2+] transients persisted in BHB-conditions (right, upper trace) but was sensitive to CdCl2 (lower trace, 100 μm). [Modified from Kirmse et al. (2010)]. **(C)** Energy substrate (DL-BHB, 4 mM) do not affect *E*_GABA/Gly_ in lumbar spinal cord motoneurons (P0–P5 rats). IPSPs evoked by electrical stimulation of ventral funiculus (*vertical arrows*) at different holding potentials in MNs from P4 rat in control and DL-BHB (4 mM) conditions. Right panel demonstrates the amplitude of IPSPs as a function of holding potential in both control and DL-BHB conditions, respectively. [Modified from Bos and Vinay (2012)]. **(D)** Energy substrates (DL-BHB, 4 mM) do not affect GABA-mediated depolarization in cultured hippocampal neurons. Average muscimol (10 mM)-mediated responses in control and BHB (4 mM) conditions (left). The peak response of the muscimol-mediated depolarization was not affected by BHB. [Modified from Waddell et al. (2011)]. **(E)** Energy substrates (pyruvate, 5 mM) do not affect *E*_GABA_ in Xenopus tadpole optic tectum. Example of Evoked GABA response recorded in gramicidin-perforated patch clamp configuration at different holding potentials (left traces) and corresponding I/V relationship (middle graph). *E*_GABA_ observed in cells recorded at two different developmental stage in control and pyruvate (5 mM) conditions. Horizontal bars show the average *E*_GABA_ values (right). [Modified from Khakhalin and Aizenman (2012)].

The Zilberter group published a set of papers suggesting that the availability of ESs determines GABA polarity in neonatal brain slices (Holmgren et al., 2010; Zilberter et al., 2010; Mukhtarov et al., 2011). According to these authors, GABA is hyperpolarizing and inhibitory, and GDPs are not detected in neonatal slices exposed to oxidative or complementary ESs, consisting of adding to glucose the ketone body metabolite dl-β-hydroxybutyric acid (BHB;4 mM), lactate (4–5 mm), or pyruvate (4–5 mM) to artificial cerebrospinal fluid (ACSF). These ESs are suggested to modulate GABA polarity by improving metabolic needs. The authors also reported that the modulation of GABA polarity does not depend on an acidification expected to occur with these weak intracellular acids (Mukhtarov et al., 2011). However, these observations have been contradicted by many groups relying on a wide-range of preparations and techniques (Figure 1):

1. Using the same preparation, experimental apparatus, and concentrations (Tyzio et al., 2011), BHB like physiological concentrations of lactate or pyruvate do not alter the depolarizing and excitatory actions of GABA in immature slices (Figure 1A). These results were verified using multiple recording techniques including dual single channel recordings of NMDA and GABA receptors, perforated- and whole-cell patch clamp recordings, and field recordings. Also, non-invasive recording using dynamic two-photon imaging of hundreds of neurons revealed that the generation of GDPs is unaltered by BHB, but fully blocked by an antagonist of the chloride importer NKCC1 confirming that neurons in immature slices have high intracellular Cl. Also physiological levels of pyruvate and lactate had no effects on the excitatory actions of GABA or on the generation of GDPs (see below). It is worth noting to stress that immature neurons are extremely resistant to anoxia or aglycemia (Cherubini et al., 1989; Krnjevic et al., 1989) and reliable evidence that neurons lack energy in glucose 10 mM remains to be shown.
2. Using minimally invasive optical and electrophysiological techniques (calcium imaging, cell-attached single K^+^ channel and loose patch recordings), Kirmse and collaborators reported that BHB does not affect GABA depolarization in cortical plate neurons from immature slices (Kirmse et al., 2010) (Figure 1B). It is important to note that the perfusion rate used in this study was identical to that used by Zilberter and colleagues (Holmgren et al., 2010), challenging the suggestion that the discrepancy is due to a slowing of the perfusion rate that weakens spontaneous activity through a lack of energy (Bregestovski and Bernard, 2012). Moreover, reducing the perfusion flow rate inhibits spontaneous activity independently of energy deprivation in the embryonic hindbrain-spinal cord and in the postnatal cortex *in vitro* (Yvert et al., 2011).
3. Bos and Vinay showed that in the neonatal rat lumbar spinal cord *in vitro* supplementing the ACSF with physiological concentrations of ESs does not alter the reversal potentials of synaptic currents mediated by GABA and glycine, known to be depolarizing at early developmental stages (Bos and Vinay, 2012) (Figure 1C). Moreover, ESs did not affect the depolarizing action of GABA on primary afferent terminals.
4. Using an electrophysiological and imaging approach Waddell and collaborators showed that the addition of BHB to cultured hippocampal neurons does not affect neuronal Ca^2+^ nor GABA_A_-mediated membrane depolarization (Waddell et al., 2011) (Figure 1D). These observations confirm extensive observations by Poo, Woodin and many other laboratories (Ganguly et al., 2001; Chudotvorova et al., 2005; Fiumelli et al., 2005; Khirug et al., 2005; Balena and Woodin, 2008; Yeo et al., 2009; Succol et al., 2012), showing that the developmental shift of GABA actions occur in cultures where abundant energy is supplied.
5. Using isolated brains of developing wild-type *Xenopus* tadpoles, Khakhalin and Aizenman demonstrated that glucose-based ACSF is an appropriate extracellular media for *in vitro* studies and that pyruvate does not alter GABA polarity (Khakhalin and Aizenman, 2012) (Figure 1E). They also showed that the more immature neurons found in the vicinity of the central are more likely to show depolarizing responses to GABA than mature ones.
6. Kaila and colleagues have invalidated the metabolic interpretation of the actions of lactate on immature slices (Ivanov et al., 2011; Mukhtarov et al., 2011). Using extracellular recordings of GDPs, while monitoring the mitochondrial membrane potential and intracellular pH, they demonstrated that standard glucose concentrations do not alter mitochondrial energy metabolism, and are thus an adequate energy supply for neonatal neurons in acute slices (Ruusuvuori et al., 2010). Supplementing the standard physiological solution with l-lactate did not produce a change in mitochondrial membrane potential, whereas withdrawal of glucose, in the presence or absence of l-lactate produced a pronounced depolarization. Furthermore, d-lactate (a poor substrate of mitochondrial metabolism) caused a prompt inhibition in GDP frequency similar to l-lactate, excluding a metabolic explanation of the shifts. The suppression of GDPs was strictly proportional to the fall in pH_i_ caused by weak carboxylic acids (l-lactate, d-lactate, or propionate) or by an elevated CO_2_. These observations clearly indicate that when the effects of ESs are observed, they are due to a change in intracellular pH, and not to a decrease in mitochondrial energy metabolism.
7. In acutely isolated retinas, Barkis and collaborators demonstrated that the percentage of retinal ganglion cells in which the GABA agonist muscimol increased somatic Ca^2+^ was not altered by pyruvate (Barkis et al., 2010).

Therefore, seven different studies have refuted the results of the Zilberter and colleagues and to the best of our knowledge there is not a single study confirming their findings. In addition, the effect of modifying the metabolic supply of the ACSF has been tested in human epileptic slices (G Huberfeld, personal data). The neocortex surrounding gliomas and the subiculum deafferentated by the hippocampal sclerosis generate spontaneous interictal discharges *in vitro*. These activities are sustained by depolarizing effects of GABA related to abnormally high Cl^−^ concentration in some pyramidal cells, so that re equilibrating intracellular Cl^−^ by low amounts of the diuretic bumetanide controls the epileptic discharges. Replacing 5 mM of glucose by 5 mM pyruvate (*n* = 2) or 5 mM lactate (*n* = 2) in the ACSF did not affect interictal-like discharges both in the cortex and the subiculum. Therefore, the depolarizing actions of GABA are valid in different preparations extending from tadpoles to humans.

Interestingly, the criticism of the developmental shift is quite restricted to the post-natal period. Indeed, referring to the study of Wang and Kriegstein (2008). Bernard and Bregestovski state “*Treatment of mice with bumetanide during the period of embryonic cortical developmental results in disruption of excitatory synapse formation* (Wang and Kriegstein, 2008). *As bumetanide antagonizes the Na^+^_−_ − K^+^ − 2Cl^−^ cotransporter (NKCC1), which accumulates intracellular chloride, these observations suggest that Cl in embryonic neurons is elevated and plays an important signaling role in developmental processes.”* Therefore, the authors agree that GABA must depolarize embryonic neurons confirming the developmental sequence but challenge only its time course with a suggested shift around delivery (see below). Yet, in the same study, Wang and Kriegstein showed that bumetanide exerts its most dramatic effects when “*administered between E17 and P7” but not when it is only administered in utero* indicating that GABA must depolarize neurons during the first postnatal week and not only in the embryo. Therefore, the studies these authors rely upon to develop their arguments invalidate their conclusions confirming both the sequence and the timing reported previously.

There are three other concerns with the model proposed:

1. The concentrations of pyruvate (4–5 mM) used by these authors are several orders of magnitude higher than those observed in physiological conditions (20–40 fold) and never observed in the brain except under severe metabolic disorders. There are thousands of publications (2449 citations in PubMed under “pyruvate and brain disorders” as of June 2012) addressing questions related to high pyruvate as an indication of suffering of central neurons and an invitation to intervene in emergency to avoid brain complications. Collectively, these studies contradict the physiological relevance of such high doses of ESs and the suggestion that the use of pyruvate or lactate may be “therapeutically useful to treat the cause and not the consequences of neurological disorders” (Zilberter et al., 2010). Clearly, the effects of high pyruvate or lactate are due to pH shifts that directly impact intracellular chloride and not to metabolic improvements.
2. The argument that ketone bodies metabolites mimic maternal milk and are therefore more physiological than glucose cannot be reconciled with the observation that the depolarizing shift occurs in non-mammalian species including zebrafish, turtles, chicken or tadpoles (Sernagor et al., 2003; Leitch et al., 2005; Akerman and Cline, 2006; Gonzalez-Islas et al., 2009, 2010; Zhang et al., 2010). It is also incompatible with the observation that during delivery under the peak of oxytocin actions, GABA hyperpolarizes neurons in glucose perfused slices (Tyzio et al., 2006).
3. The anticonvulsive action of the Ketogenic diet is not mediated by GABAergic signals. Indeed, comparing the actions of functional and blocked ketogenesis on seizures produced by fluothryl, Minlebaev and Khazipov reported that diazepam exerted its usual anticonvulsive actions in both conditions whereas bumetanide had no significant anticonvulsive effects in both conditions (Minlebaev and Khazipov, 2011).

## The art of slicing

In spite of obvious limitations (i.e., neurons with damaged axons and dendrites, lack of long-range connexions, artificial conditions etc.) acute brain slices have provided essential information on the basic mechanisms of neuronal activity, synaptic function, network synchronization, and neuronal plasticity in both physiological and pathological conditions. While it is important to acknowledge the limitations of *in vitro* preparations, and use *in vivo* options where possible, there is still no other preparation that has enabled us to gain as much information, notably when coupled with genetic and labeling techniques to identify the operation of specific neuronal types and their roles in brain networks.

**Figure 2:**
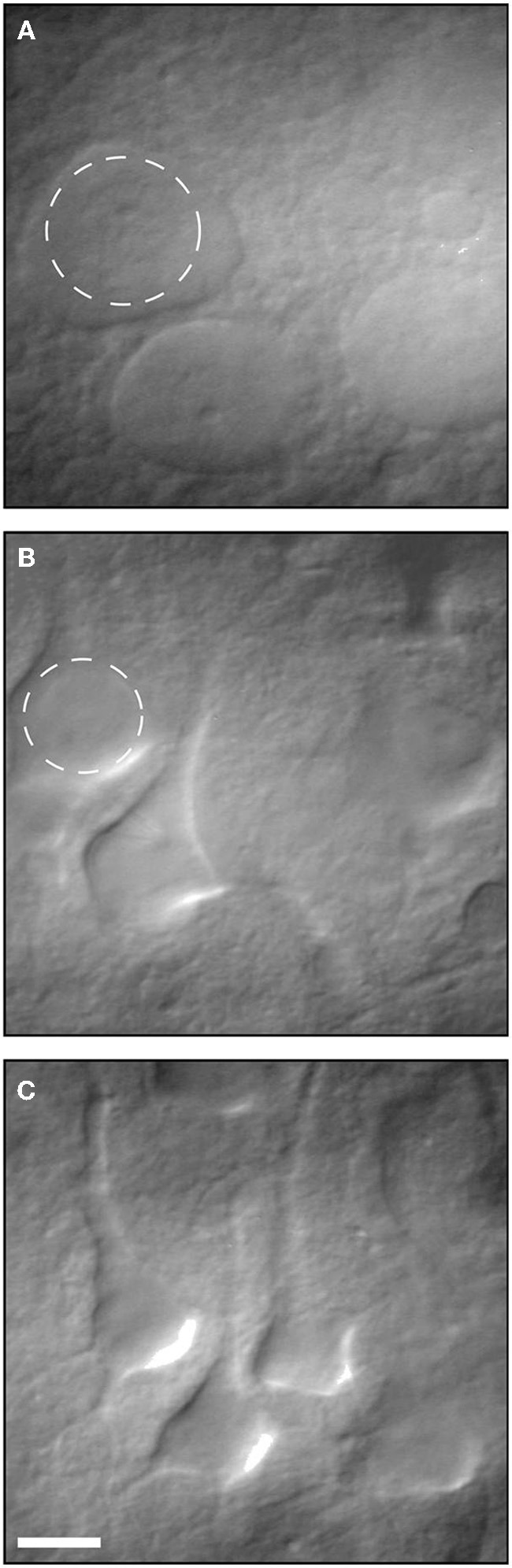
**Infrared differential interference contrast (IR-DIC) video micrographs of neurons in 400- μm-thick brain slices. (A)** Several examples of dead neurons. **(B)** Several examples of unhealthy neurons. **(C)** Examples of healthy patchable neurons. Reproduced with permission from Moyer and Brown (2002).

There is little doubt that during the slicing procedure, some neurons are damaged and slices are occasionally not fit. This has been known for decades and well-trained electrophysiologists can readily identify damaged neurons relying on their morphology and resting membrane potential. As Moyer and Brown state in the highly cited chapter on preparing brain slices for patch clamp recordings (Moyer and Brown, 2002) “*Dead neurons are always present within the first 25 μm below the surface of a brain slice.*” They recommend recording from neurons between 70–140 μm below the slice surface. Moreover, they explain in detail how visually healthy and patchable neurons are easy to distinguish from dead or unhealthy neurons using IR-DIC optic (Figure 2) and relying on their resting membrane potential. This chapter, which is highly cited, suggests that the claim that most electrophysiological studies are performed on surface layers (30–80 μm) with large amount of damaged cells is unfounded.

To challenge the physiological relevance of the excitatory actions of GABA in immature neurons, the authors of the “hypothesis and theory article” rely extensively on a recent study by Staley et al. (Dzhala et al., 2012) that used Clomeleon genetic mice to measure chloride, and extracellular multi-unit activity to measure the effects of GABA analogs. The authors of this study show that in P5–P7 slices, but not in the intact hippocampal preparations developed by Ben-Ari and colleagues (Khalilov et al., 1997), the slice surface is damaged and neurons are silent, with high intracellular chloride and no response to GABA analogs. The resting membrane potential of these “damaged neurons” was not determined and the neurons neither identified nor reconstructed. This “silent zone” shifts from 30–60 μm at P5–7 to 70–90 μm by the end of the fourth week, and this is suggested to underlie the generation of GDPs by the immature but not the adult network. However, in a follow up to this work, using the same techniques, the same group has shown that before the P5–P7 period “GABA excites immature hippocampal neurons during the early postnatal period both in the preparations of the intact hippocampus and slices and these excitatory actions are not due to the neuronal injury during slice preparation” (Valeeva et al., 2012). Therefore, again the developmental shift of the polarity of GABA actions is confirmed in rats and mice but Staley et al. suggest that the shift may take place around P5–P7. This timing is identical to that proposed already in 1989 for hippocampal pyramidal neurons (Ben-Ari et al., 1989) and repeatedly confirmed since then with a wide range of invasive and non-invasive recordings techniques (Ben-Ari et al., 2007). Therefore, it seems unreasonable to rely on the observations of Dzhala et al. (2012) to challenge the developmental sequence and its timing.

**Figure 3:**
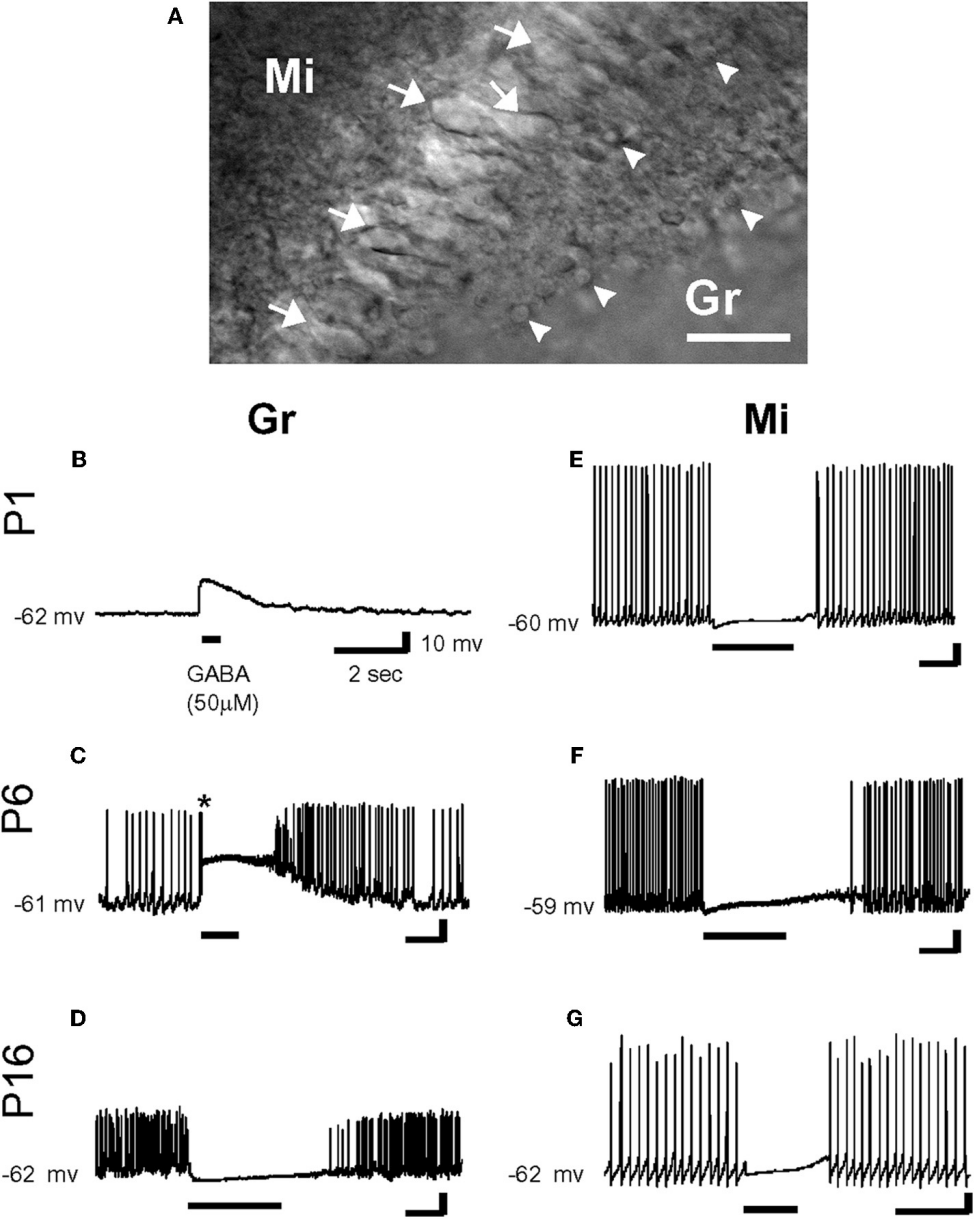
**Effects of γ-aminobutyric acid (GABA) in the developing rat olfactory bulb neurons. (A)** IR-DIC images of granule cells (Gr: arrow head) and mitral cells (Mi: arrow) at P16. Thus, olfactory bulb neurons were distinguished by their location and morphology. Scale bar: 50 μm. **(B–G)** By gramicidin-perforated patch-clamp recordings in the current-clamp mode, GABA (50 μm) depolarized P1 and P6 granule cells (**B** and **C**), and hyperpolarized P16 granule cell **(D)**. Note that GABA application evoked an action potential firing in a granule cell (* in **C**). In contrast, mitral cells were hyperpolarized by the application of GABA at any age **(E–G)**. Resting potentials are indicated at left. [From Wang et al. (2005)].

**Figure 4:**
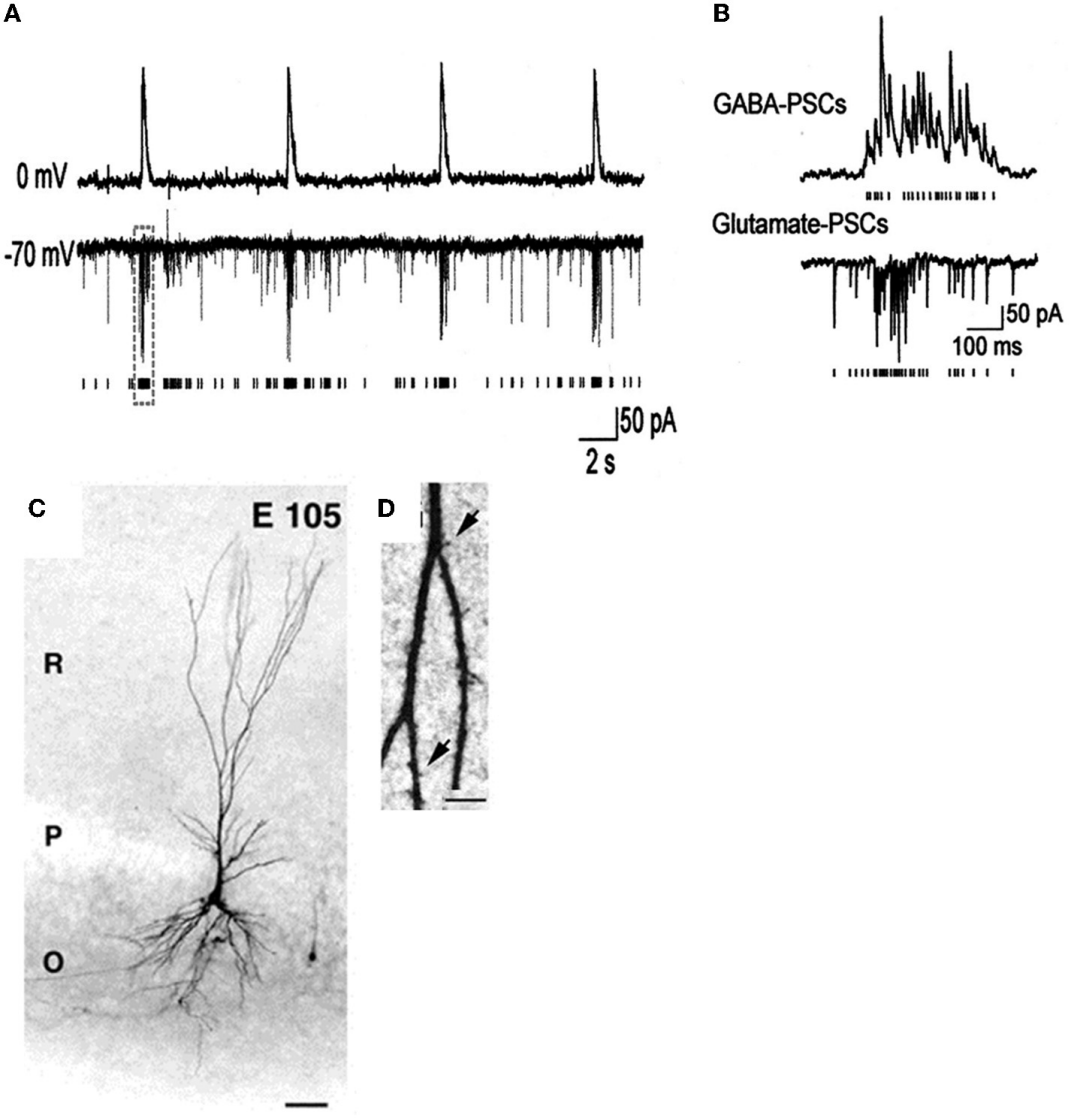
**Giant depolarizing potentials synchronize most of the Primate hippocampal neuronal activity *in utero*. (A,B)** Pair recordings of CA3 pyramidal cells and interneurons (E109). **(A)** The pyramidal cell (top trace) is recorded in whole-cell mode at 0 mV, and the interneuron (bottom trace) is recorded in whole-cell mode at the reversal potential of the GABA(A) PSCs (−70 mV) so that the AMPA PSCs are inwardly directed. Each AMPA PSC detected in the interneuron is shown as a bar below. **(B)** GDPs outlined by dashed boxes on **A** are shown on expanded time scale. Note an increase in the frequency of the GABA(A) and AMPA PSCs during GDPs. **(C)** Photomicrographs of biocytine-labeled pyramidal cells at E105. Scale bars: 50 μm. **(D)** High magnification of dendritic processes. Scale bars: I, 5 μm. [Modified from Khazipov et al. (2001)].

There is little doubt that neuronal damage can be associated with an accumulation of intracellular chloride and a shift of polarity of the actions of GABA, as reported in a wide range of insults (see below). This however neither implies that the observations made in slices are due to damage nor does it explain why the damage would be translated to depolarizing GABA at the end of the first postnatal week but neither before nor after. By measuring the resting membrane potential and other intrinsic parameters trained, electrophysiologists can readily dissociate healthy from damaged neurons. Interestingly, the original demonstration of depolarizing actions of GABA (Ben-Ari et al., 1989) relied on intracellular, not patch-clamp, recordings excluding *de facto* recording from surface neurons. Since slices are extensively used to dissect neuronal mechanisms, we would like to add the following additional points concerning the studies by Dzhala et al. (2012):

1. The authors pooled data from CA3 and CA1 hippocampal regions, pyramidal neurons and interneurons and male and female mice and male rats in spite of the well-known differences in developmental sequences that are age and neuronal type and sex dependent (Ben-Ari et al., 2007). In addition, when single neurons are functionally determined in terms of their synaptic currents and reconstructed after dye injections, they have normal features without the varicosities observed by Dzhala et al. (Tyzio et al., 1999; Khazipov et al., 2001; Hennou et al., 2002; Gozlan and Ben-Ari, 2003). The use of long lasting laser excitation can damage neurons possibly adding to the slicing paradigm (see Quilichini et al., 2012).
2. These authors used the ratiometric and genetically-encoded Cl^−^ indicator Clomeleon (Kuner and Augustine, 2000). In addition to the strong pH sensitivity of this sensor, Clomeleon has a very low sensitivity to Cl^−^ with an EC_50_ of more than 160 mM (Kuner and Augustine, 2000; Bregestovski et al., 2009). With an average *K*_D_ of 91 mM, *R*_max_ of 1.026 and *R*_min_ of 0.268 the intracellular [Cl^−^] measurements given by Dzhala et al. (2012) should be taken with caution; this is reflected by the extreme heterogeneity of reported chloride levels (from 1 to 120 mM between P5 and P7). It does not come as a surprise that neurons at the slice surface, in particular pyramidal neurons with long apical dendrites, may be damaged in their dendritic compartment.
  However, from a three dimensional perspective, neurons deep in the slice also maintain extensions above and below the slice. In fact numerous studies from acute slices have provided an excellent preservation of the dendritic tree [e.g., (Staiger et al., 2004)] when slices are prepared and cut in a manner, which leaves even long apical dendrites of layer 5 neocortical pyramidal neurons intact (Frick et al., 2008). Contrary to their interpretation, Nabekura and collaborators recorded from acutely dissociated neurons, which are more severed than in slices, yet GABA produced a hyperpolarization in most contralateral side neurons (Nabekura et al., 2002). In contrast, neurons from the ipsilateral side that were axotomized 1–3 days earlier showed GABA-induced depolarization and neurons freshly axotomized before recording were healthy enough to be hyperpolarized by GABA suggesting chronic not acute effects of lesions on Cl^−^. Interestingly, in coronal olfactory bulb slices, using gramicidin-perforated patch-clamp recordings, Wang and collaborators found that mitral cells (acutely axotomized but expressing KCC2) were hyperpolarized and inhibited by GABA, while granule cells (not axotomized but lacking KCC2) were depolarized and/or excited by GABA (Wang et al., 2005) (Figure 3) [also see Toyoda et al. (2003)]. Both types of cells were located at similar depth in the slices, illustrating the cell type dependence of the polarity of GABA and contradicting the suggestion that damage underlies the depolarizing actions of GABA (Bregestovski and Bernard, 2012; Dzhala et al., 2012). Similarly, many studies in which immature surface and deep neurons were recorded and reconstructed were found to have healthy resting membrane potentials with neuronal arbors without signs of varicosities or swelling (review in Ben-Ari et al., 2007).
  Furthermore, Cajal-Retzius cells in the marginal zone/layer I of the cerebral cortex of immature mammals project their dendrites exclusively in this upper cortical layer where most of the axonal projections are located making this cell type an ideal candidate to study GABA actions in tangential neocortical slices. Experimental studies and a mathematical model provide strong evidence that the steady-state [Cl^−^]i of these cells is approximately 30 mM and that activation of GABA-A and glycine receptors in this cell type has an excitatory action under experimental conditions which mimic the *in vivo* situation (Kilb et al., 2002; Achilles et al., 2007; Kolbaev et al., 2011a,b). Indeed, similar prominent on-going spontaneous network activity is observed in the somatosensory cortex of newborn rats *in vivo* (Yang et al., 2009, 2012).
3. Dzhala et al. (2012) suggest that superficial neurons are functionally disconnected from the network in mature but not immature slices because in the former, neurons are completely dead whereas in the latter, superficial neurons are damaged but can still contribute to network activity. This argument is speculative and lacks experimental evidence. Moreover, the distribution of the damaged neurons with respect to slice depth differs from the depth distribution of neurons excited by GABA, as only neurons of the core of the slice were hyperpolarized by GABA (see Figure 6F of Dzhala et al., 2012) casting some doubt on the relationship between damage and GABA responses. It is quite difficult to understand why surface neurons would exhibit depolarizing GABAergic responses and alter spontaneous network activity between P5–P14 and not earlier or later. This is also inconsistent with the observation that during the 2nd postnatal week, GABA exerts hyperpolarizing actions and the GDPs are replaced by Large Hyperpolarizing Potentials at that stage (Ben-Ari et al., 1989).
4. The damaged tissue hypothesis is difficult to reconcile with the transient and abrupt shift of GABA from the depolarizing to the hyperpolarizing direction triggered by maternal oxytocin just before parturition that exerts a protective and analgesic action (Tyzio et al., 2006; Mazzuca et al., 2011). It is also difficult to reconcile with the accelerated transition of GABA action from excitation to inhibition, observed in hippocampal slices from mice reared during the first two postnatal weeks in an enriched environment (He et al., 2010), the negative shift in E_GABA_ caused by maternal separation-stress (Galanopoulou, 2008a,b), the sex dependence of the timing of the shift (Galanopoulou et al., 2003; Galanopoulou, 2008a,b) or the neuronal type specific effects of GABA (Banke and McBain, 2006; Rheims et al., 2008). It is also difficult to reconcile with the delayed shift of GABA action observed in neuronal cultures (Ganguly et al., 2001; Barkis et al., 2010) and in the retina from dark-reared turtles (Sernagor et al., 2003). If immature brain slices are severely damaged, it is difficult to understand why adult slices that are more susceptible to energy deprivation would not generate GDPs. Similarly, it is quite unlikely that all the studies reporting depolarizing actions of GABA were made form surface damaged neurons.
5. Bregestovski and Bernard (2012) consider GDPs to be a pattern generated by the energy insufficiency of glucose perfused slices, reminiscent of inter-ictal activities observed in epileptic or damaged preparations. However, GDPs have been recorded in embryonic macaques (Khazipov et al., 2001) (Figure 4) at times when Bernard and Bregestovski agree that GABA is genuinely depolarizing (see below) and cannot be due to damage. Moreover, GDP-like activity has been recorded *in vivo* in freely moving rats (Leinekugel et al., 2002) and organotypic slice cultures (Mohajerani and Cherubini, 2005). In contrast to interictal events, GDPs that are restricted to interneurons at an early stage (Ben-Ari et al., 2007), propagate form the septal to the caudal poles of the hippocampus (Leinekugel et al., 1998), have a intracellular chloride dependent reversal potential (Khazipov et al., 1997) and are blocked *in vivo* and *in vitro* by pharmacological or genetic invalidation of the chloride importer NKCC1 (Sipila et al., 2006; Nardou et al., 2011; Takano et al., 2012).
  The depolarizing actions of GABA and the presence of correlated network activity have been observed in a wide range of spinal cord and brain tissue preparations that are not subject to neuronal damage or energy deprivation. These include: brain slabs (Owens et al., 1996), the relatively intact chick, mouse, and rat embryonic spinal cord preparations (Landmesser and O'Donovan, 1984; Chub and O'Donovan, 2001; Hanson and Landmesser, 2003; Ren and Greer, 2003; Yvert et al., 2004; Delpy et al., 2008; Gonzalez-Islas et al., 2009) and the intact retina (Fischer et al., 1998). Even if neuronal cultures have some limitations and re-establish a neuronal network that differs from the *in vivo* one, they are not damaged and show a complete axonal and dendritic arborization. Nonetheless, the developmental shift in GABAergic responses and KCC2 expression has been observed in neuronal cultures (Obrietan and van den Pol, 1995; Chudotvorova et al., 2005; Khirug et al., 2005; Mohajerani and Cherubini, 2005; Balena and Woodin, 2008; Yeo et al., 2009; Succol et al., 2012) where GDPs have been recorded with similar features to slice GDPs (Voigt et al., 2001). To test whether the depolarizing actions of GABA in developing neurons *in vitro* might be due to the damage induced by culturing the cells, rat neurons from embryonic day 15 and from postnatal day 5 were compared after 1 day *in vitro*. As determined with gramicidin-perforated patch recording, the chloride reversal potential (E_Cl_) was −38.6 mV for the embryonic neurons, whereas the 12-day older postnatal cells showed an E_Cl_ of −57.4 mV (Chen et al., 1996), a difference of almost 20 mV. If a positive E_Cl_ was due primarily to neuronal damage, then one would expect the older cells in which axon and dendrite injury would be more severe, to show the more depolarized E_Cl_, but this was not seen. In addition, transfection of the outward Cl^−^ transporter KCC2 gene into developing neurons that normally have depolarizing GABA responses shifts E_GABA_ in a negative direction and causes GABA hyperpolarization, in keeping with the developmental sequence of chloride co-transporters (Chudotvorova et al., 2005; Bortone and Polleux, 2009). All these studies indicate that the depolarizing action of GABA is not simply due to injury from slicing.
  In addition, *in vitro* studies on the retina use the whole mount preparation. In this preparation, the whole retina is isolated from the eye by sectioning the optic nerve (which consists of the axons of all ganglion cells) and flattened for recording. Hence, although one could argue that these cells are indeed axotomized, the rest of the network is virtually intact, except for a few radial incisions made to flatten the tissue. Despite these radical differences with brain slices, GABA_A_ responses are depolarizing/excitatory when the retina is immature, and they switch to their mature hyperpolarizing/inhibitory action very abruptly, especially in mammals. Indeed, pressure applying GABA_A_ agonists on the mouse ganglion cell layer depolarizes these cells until P6, and this effect disappears virtually overnight (Zhang et al., 2006). By P7, the same experimental procedure does not depolarize ganglion cells anymore. Hence, it is difficult to reconcile these observations with the argument that depolarizing GABA is an artefact due to tissue slicing and/or metabolic stress.
6. If surface damaged neurons require more energy than deeper healthier ones, GABA should depolarize surface neurons in glucose media and hyperpolarize them when ESs are added. It is not reasonable to base a theory on two studies that are incompatible. Indeed, in the study of Zilberter and colleagues (Holmgren et al., 2010), GABA excites all neurons in a slice because they are energy deprived in glucose-perfused conditions; whereas in the study of Dzhala et al. (2012) performed in glucose-perfused slices, only surface neurons that are damaged are depolarized by GABA: yet deeper ones should be energy deprived as they are perfused with glucose! If Zilberter and colleagues show in the future that surface neurons can be rescued by adding ESs, this would invalidate the conclusions of Dzhala et al. (2012) according to which the damage produced by the slicing procedure, but not the lack of energy substrate, is the cause of depolarizing GABA. One cannot base a model on two incompatible observations: this contradiction precludes a unifying concept.
7. Using *in vitro* hypothalamic slices, Yarom and collaborators have shown that GABA acts in the supra-chiasmatic nucleus as an inhibitory neurotransmitter at night and an excitatory one during in daytime (Wagner et al., 1997). It is difficult to reconcile these features with an energy deprivation and/or surface damage explanation.
8. Depolarizing GABA action persists in newborn neurons in the adult neurogenic zones. In the subventricular zone of neonatal and young adult mice, GABA depolarizing action exerts an important role in the migration and maturation of newborn neurons (Gascon et al., 2006), and the proliferation of neural progenitor cells, a different cell type where GABA is also depolarizing (Bolteus and Bordey, 2004; Young et al., 2010; Taylor et al., 2011). In the young SVZ-olfactory bulb, GABA action shifts from depolarizing to hyperpolarizing between week 2 and 3 after cell birth. This shift is important for cell dendritogenesis during a critical period of cell synaptic integration and plasticity (Taylor et al., 2011). In the adult hippocampal neurogenic zone, GABAergic excitation promotes neuronal differentiation, synaptogenesis, and integration of progenitors into the existing network following a scheme similar to that observed early in postnatal developmental (Ge et al., 2006). Thus, in neonatal as well as adult glucose-perfused slices, newborn olfactory bulb and hippocampal granule cells have depolarizing GABA. How does this fit with an energy supply explanation? Why would the lack of energy supply by glucose affect newborn but not adult neurons in the same slice? In addition, why would newborn olfactory neurons develop GABA before glutamate responses and synapses and rely upon excitatory GABA during their maturation (Carleton et al., 2003; Platel et al., 2008).

## Synchronized activities in immature neurons and actions of GABA receptor antagonists

**Figure 5:**
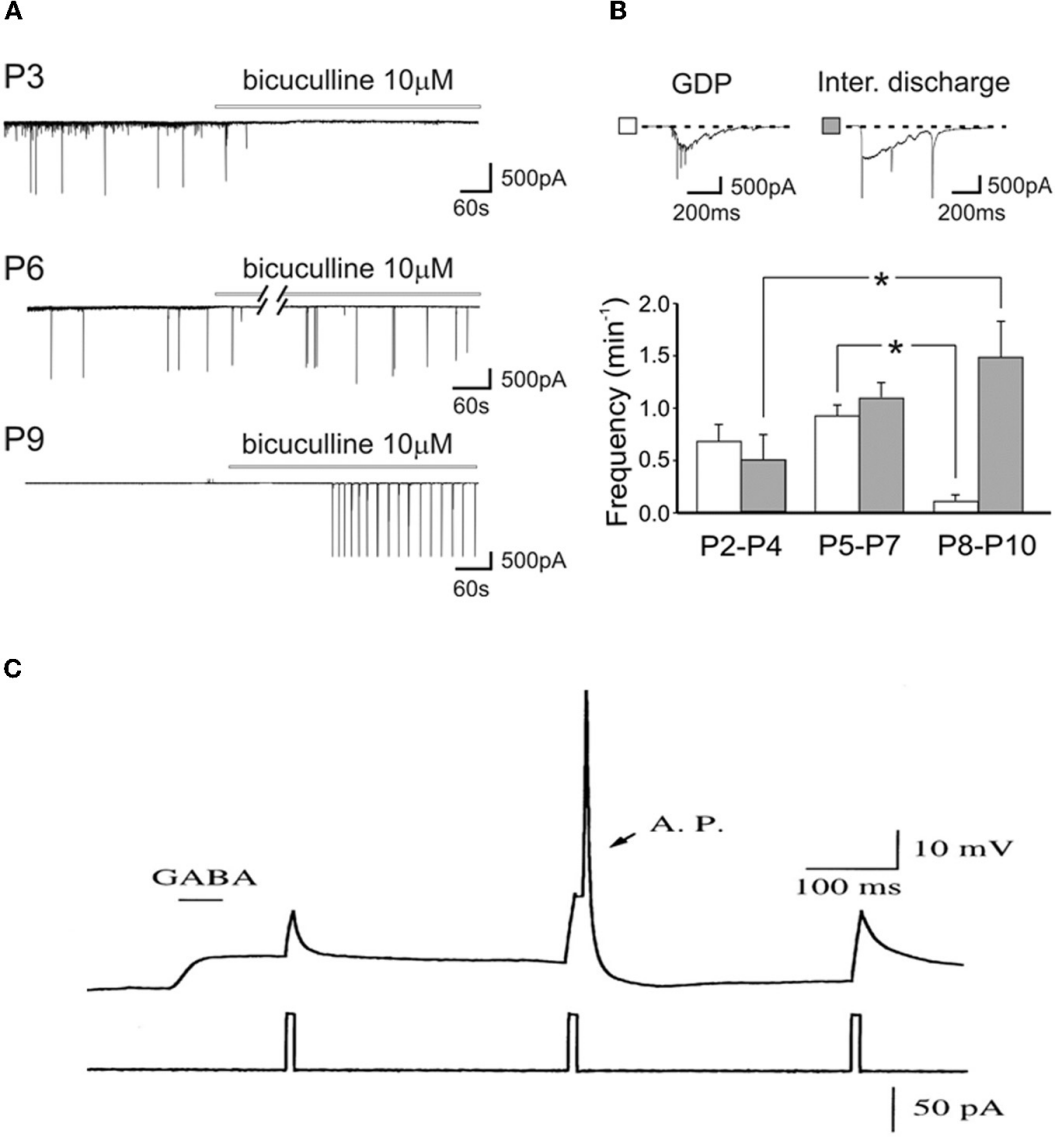
**Dual actions of GABA in the developing brain. (A)** Representative traces recorded from CA1 pyramidal neurons in hippocampal slices obtained at P3, P6, and P9, in control conditions and during bath application of bicuculline (bars). Note the disappearance of GDPs with bicuculline at P3 but not at P6 and P9. **(B)** Frequency histograms of GDPs (white columns) and interictal bursts (gray columns) recorded at different times of postnatal development. Insets above the graph: a giant depolarizing potential (GDP) and an interictal discharge shown on an expanded time scale [Modified from Le Magueresse et al. (2006)]. **(C)** Relative timing determines whether depolarizing GABAergic response are inhibitory or excitatory. A sub threshold depolarization, induced by positive current injection, can be shunted when applied near the peak of the GABA response, or can trigger an action potential (AP) when applied at later time during the GABAergic response. (gramicidin-perforated whole cell recording of hypothalamic neurons, from E15–E18 Sprague-Dawley rats, cultured for 2–5 days *in vitro*). [Modified from Gao et al. (1998)].

As GABA_A_ receptor antagonists generate inter-ictal discharges in neonatal slices, and trigger seizures in pup rats (Baram and Snead, 1990), Bregestovski and Bernard (2012) conclude that GABA must be inhibitory at that age. However, already in the pivotal 1989 paper, Ben-Ari et al. showed that GABA receptor antagonists first block ongoing activity and then generate seizures (Ben-Ari et al., 1989). It is a common observation that, in pups, bicuculline-induced inter-ictal discharges develop very slowly (often in min) and in comparison with juvenile animals occur at very low frequency and their frequency increases with age (Le Magueresse et al., 2006) (Figures 5A,B). The increase of inter-ictal discharges with age is associated with a concomitant decrease in frequency of GDPs an effect that cannot be explained by tissue damage. The *in vivo* actions are not surprising since intracellular chloride gradients are different between different cells and different brain structures. In the spinal cord, the shift of GABA from the depolarizing to the hyperpolarizing direction occurs well before the forebrain. In some interneurons synaptic GABA may already be inhibitory at birth (Banke and McBain, 2006) in keeping with their earlier development in comparison to pyramidal neurons (Tyzio et al., 1999; Hennou et al., 2002; Gozlan and Ben-Ari, 2003). As repeatedly stressed, GABA exerts dual actions on immature neurons exciting and inhibiting neurons at the same time even when GABA is strongly depolarizing because of the well known shunting actions of GABA (Andersen et al., 1980; Staley and Mody, 1992; Gao et al., 1998; Gulledge and Stuart, 2003) (Figure 5C).

Using isolated spinal cord preparations from chick or mouse embryos many authors have described an excitatory effect of GABA and glycine at early embryonic developmental stages. Delpy and colleagues have recently demonstrated that embryonic mouse spinal motoneurons *ex vivo* also exhibit a switch of reversal potential of the GABA-induced and glycine-induced currents from excitatory to inhibitory, this switch occurring after E15.5 (Jean-Xavier et al., 2006; Delpy et al., 2008; Stil et al., 2009). Interestingly, this maturation parallels the appearance of left and right alternated locomotor activities in mouse (Branchereau et al., 2000) and rat (Kudo et al., 2004). It is now established that chloride-mediated excitation play a crucial role in the genesis of the spinal cord spontaneous activity both in the chick and in the mouse embryo (O'Donovan, 1999; Branchereau et al., 2000; Hanson and Landmesser, 2003; Ren and Greer, 2003) and in promoting neurogenesis from the earliest stages of spinal cord embryonic development in the zebrafish (Reynolds et al., 2008).

GABA affects the behavior of retinal networks in an age-dependent manner during development. Indeed, the GABAA antagonist bicuculline has completely different effects on the dynamics of global spontaneous network activity (retinal waves) before and after P7 (Hennig et al., 2011), which is precisely when GABA switches polarity. Moreover, stable recordings of network activity are readily performed from the neonatal mouse retina continuously for 2–3 days, at relatively slow perfusion rate (about 1 ml/min; Hennig et al., 2011) invalidating the suggestion that these differences are due to perfusion rates (Bregestovski and Bernard, 2012).

## The KCC2/NKCC1 expression provides a mechanistic substrate to the action of GABA

**Figure 6:**
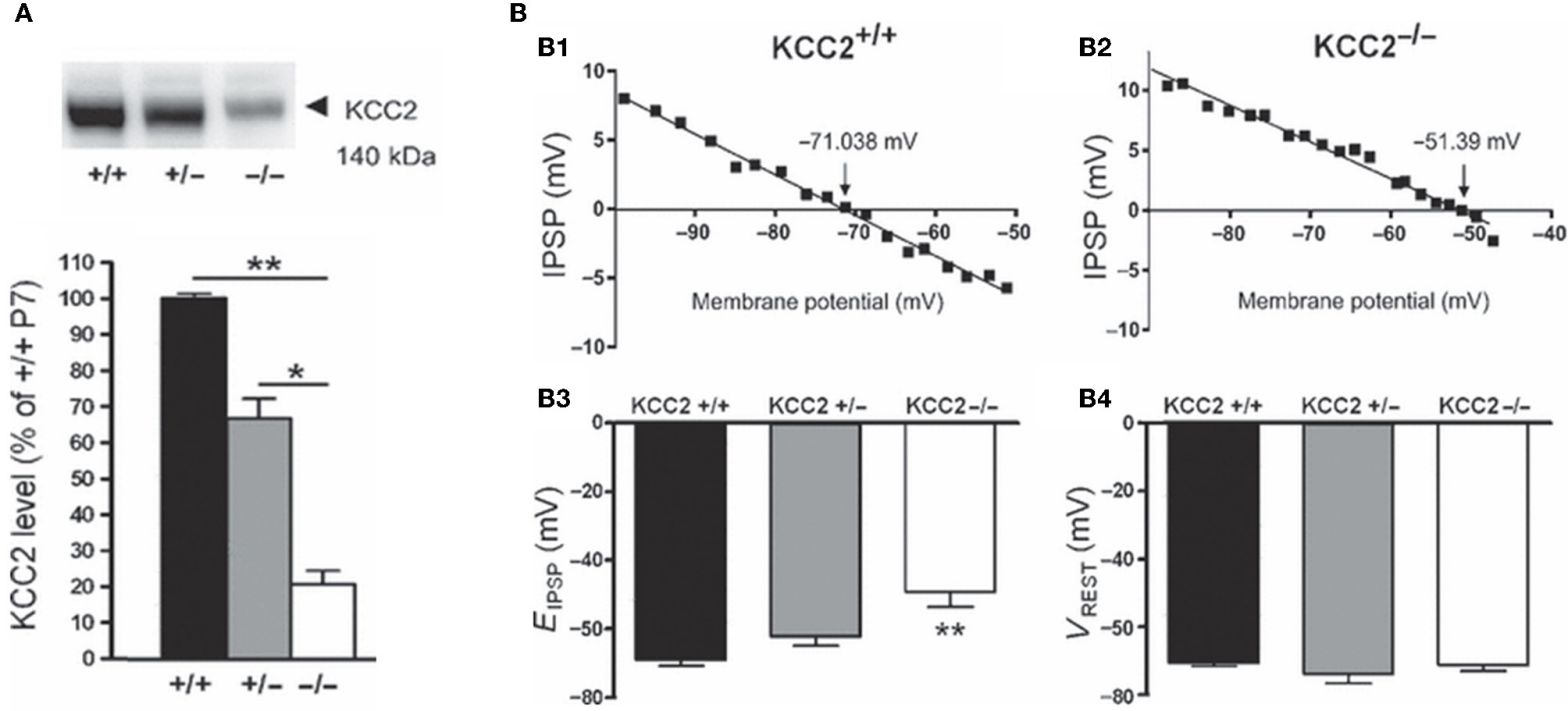
**The depolarizing action of GABA correlates with *in vivo* KCC2 expression. (A)** Western blot analysis of KCC2 expression in the lumbar spinal cord at postnatal day 7 (~140 kDa). The KCC2 expression is significantly reduced in KCC2b^−/−^ mice. **(B)** Reversal potentials of IPSPs in lumbar MNs of wild-type and KCC2 mutant mice at postnatal day 5–7. Amplitude of IPSPs plotted against holding potential giving an *E*_IPSP_ at −71.038 and −51.39 mV in a wild-type **(B1)** and KCC2b^−/−^ **(B2)** MN, respectively. The *E*_IPSP_ was significantly more depolarized in KCC2^−/−^ than in KCC2b^+/−^ and KCC2b^+/+^ **(B3)**. *V*_REST_ was similar in all gentotypes **(B4)**. [Modified from Stil et al. (2011)].

Several lines of evidence suggest that changes in chloride homeostasis are instrumental in setting the direction of GABAergic signaling (Blaesse et al., 2006). In particular, the developmentally-regulated expression of the cation-chloride importer NKCC1 and exporter KCC2 is determinant for the depolarizing action of GABA in immature neurons. KCC2 is the only member of the K^+^-Cl^−^ co-transporter gene family that is active under physiological conditions, in the absence of osmotic stress (Mercado et al., 2006; Acton et al., 2012). By extruding Cl^−^ from the neuron under isotonic conditions, KCC2 maintains a low concentration of neuronal Cl^−^, which is essential for GABA to be inhibitory. However, in many systems, KCC2-mediated Cl^−^-extrusion develops progressively during postnatal life underlying the delayed maturation of hyperpolarizing actions of GABA. During early developmental stages, KCC2 labeling is largely intracellular on the membranes of transport vesicles (Gulyas et al., 2001) and when present, its actions on spines are not mediated by alteration of chloride gradient (Li et al., 2007). Moreover, the KCC2 that is expressed in the neuronal membrane is mainly monomeric (Blaesse et al., 2006). Efficient KCC2-mediated Cl^−^-extrusion occurs when KCC2 exists in the oligomerized state (Blaesse et al., 2006; Uvarov et al., 2009; Watanabe et al., 2009). Thus, given that KCC2 expression in the neuronal membrane is low during the first postnatal week, coupled with the fact that its expression is largely monomeric, suggests that there is no mechanism for Cl^−^ extrusion at this developmental stage, and thus that GABA cannot be hyperpolarizing/inhibitory. That an up-regulation of KCC2 plays a major role in the developmental switch in GABA/glycine signaling has been repeatedly demonstrated by the use of knock-out mice. For instance, E_GABA/glycine_ is ~20 mV more positive in mice lacking the KCC2b isoform than in wild-types, at the same neonatal stage and in identical *in vitro* conditions [glucose as the sole energy substrate (Stil et al., 2011)] (Figure 6). This is confirmed in direct measures of how fast immature and adult neurons remove large chloride influxes generated by applications of pulses of GABA (Nardou et al., 2011).

In pathological conditions, including epilepsy (Cohen et al., 2002; Huberfeld et al., 2007), axonal injury (van den Pol et al., 1996; Nabekura et al., 2002), trauma and osmotic shock (van den Pol et al., 1996), neuropathic pain (Coull et al., 2005), inflammatory hyperalgesia, allodynia (Funk et al., 2008), and neurodegeneration (Lagostena et al., 2010) GABA becomes depolarizing and excitatory in adulthood as well via a change in Cl^−^ homeostasis. The injury-mediated shift to GABA depolarization is due to two independent factors; one is the immediate influx of Cl^−^ and the other may be due to a shift in gene expression that recapitulates the ontogeny of neuronal Cl homeostasis and consequently, GABA actions (van den Pol et al., 1996). How can these observations be reconciled with an energy insufficiency of glucose or a slice damage of surface neurons? In addition, several observations also indicate that a low KCC2 membrane expression contributes to the development of epileptiform discharges in adult hippocampal slices from epileptic patients (Cohen et al., 2002; Huberfeld et al., 2007). Using the triple chamber with the two interconnected hippocampi placed in their independent chambers, the formation by recurrent seizures of an epileptogenic mirror focus in the naïve contralateral hippocampus is associated with a persistent loss of KCC2 and reduced capacity of epileptic neurons to expel chloride (Nardou et al., 2011). In addition, age matched slices prepared from naïve intact hippocampi and epileptic ones generate, respectively, GDPs and interictal activities invalidating a damage explanation. Clearly, enhanced episodes of activity or seizures, acting on KCC2 (or NKCC1), alter the polarity of GABA actions via tyrosine phosphorylation mediated internalization of KCC2. This results in profound *in vivo* and *in vitro* consequences on epileptogenicity (Lee et al., 2010, 2011).

In addition, hippocampal slices obtained from adult (6 months old) mice chronically deprived of NGF, in comparison to age-matched controls, exhibit depolarizing and excitatory responses to GABA (Lagostena et al., 2010). Interestingly, these effects were found to be associated with a reduced expression of the mRNA encoding for *Kcc2* and for α7 nicotinic acetylcholine receptors (thought to control the expression of KCC2) and to play an instrumental role in the shift of GABA from the depolarizing to the hyperpolarizing direction (Liu et al., 2006; Lozada et al., 2012). Transgenic mice with decreased expression of KCC2 are more susceptible to pentylenetetrazole-induced seizures (Tornberg et al., 2005) and full KCC2 KO mice die at birth with seizures (Khalilov et al., 2011). Mice with targeted deletion of the KCCb isoform exhibit spontaneous generalized seizures and die before the third postnatal week (Woo et al., 2002). Collectively, these observations reflect the importance of early chloride regulation.

The low expression of KCC2 accounts also for synapse driven GABA_A_-mediated membrane depolarization and the burst firing observed in reticular nucleus neurons in thalamic slices from P13–P35 old mice (Sun et al., 2012). A high expression of NKCC1 accounts for the depolarizing effect of GABA in dorsal root ganglion cells in culture (isolated from 6–12 weeks-old mice; i.e., not immature) as shown by the ~20 mV negative shift of E_GABA_ in cells isolated from NKCC1 knock-out mice (Sung et al., 2000). Furthermore, the imbalance between NKCC1 and KCC2 may affect GABA action in some cell compartments but not in others as for instance in the initial axon segment where the lack of KCC2 is responsible for GABA-induced membrane depolarization in adult cortical principal neurons (Khirug et al., 2008). It is worth noting that in adult dentate granule cells, the depolarizing action of GABA is independent of the KCC2/NKCC1 unbalance but is set by the unusual hyperpolarized value of V_rest_, well below E_GABA_ (Staley and Mody, 1992; Kraushaar and Jonas, 2000; Chiang et al., 2012; Sauer et al., 2012). Nevertheless, knocking out NKCC1 in newborn hippocampal granule cells in adult, in newborn olfactory bulb neurons in neonate, and in newborn embryonic neurons led to a premature shift in GABA depolarizing action (Ge et al., 2006; Wang and Kriegstein, 2008; Taylor et al., 2011). All these observations provide compelling evidence that the depolarizing action of GABA is a fundamental property of developing systems and not due to cell injury following the slicing procedure in neonates.

## The polarity of GABA actions shifts during development in non-mammalian species *In vivo* and *In vitro*

**Figure 7:**
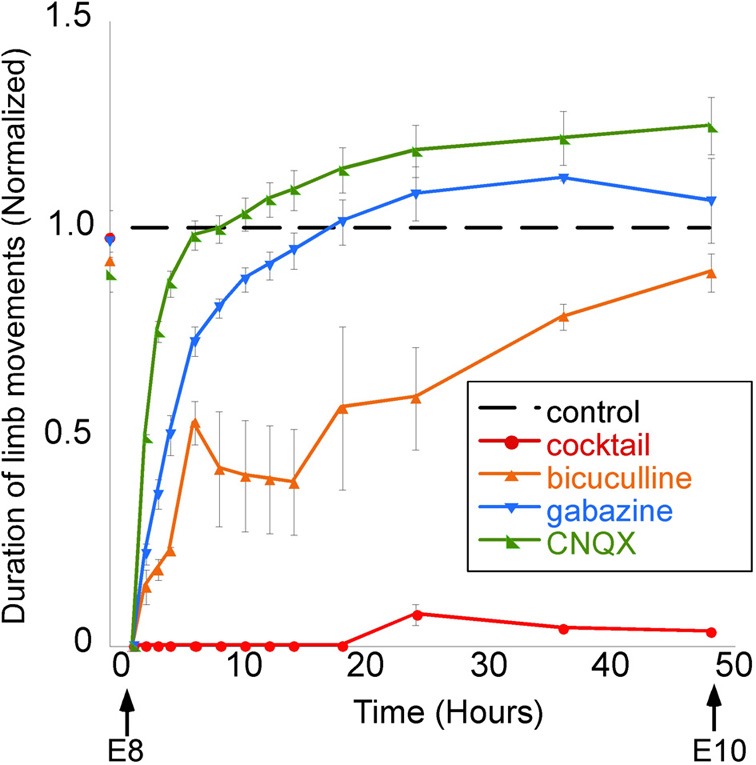
**GABAergic or glutamatergic receptors can be chronically blocked without a prolonged reduction of spontaneous network activity.** Graph of the average amount of time chick embryos moved during a 5-min period of observation, obtained once every 0.5–12 h. Data are normalized to control values at each observation time point/stage, thus producing the dotted line for comparison. Treatment with a single receptor antagonist transiently reduces embryonic movements; however, movements return to control levels within 12 h. A mixture of gabazine, CNQX, strychnine, and APV nearly abolished limb movements for 48 h. [Modified from Wilhelm and Wenner (2008)].

The proposal that GABA may not be depolarizing in developing neurons *in vivo* is further disputed by the results of studies showing that the *in vivo* application of GABA antagonists inhibits the spinally generated limb movements (Wilhelm and Wenner, 2008). In the mid-gestation embryonic chick, injecting GABA antagonists (bicuculline or gabazine) into the egg blocked or reduced the duration of spinally generated embryonic movements for several hours, which was an even more effective block than *in ovo* application of an AMPA receptor antagonist (Wilhelm and Wenner, 2008) (Figure 7). On the other hand, in the late-gestation embryonic chick, injection of bicuculline had the opposite effect (increased movements), suggesting GABA had become inhibitory at later stages (Sedlacek, 1985).

**Figure 8:**
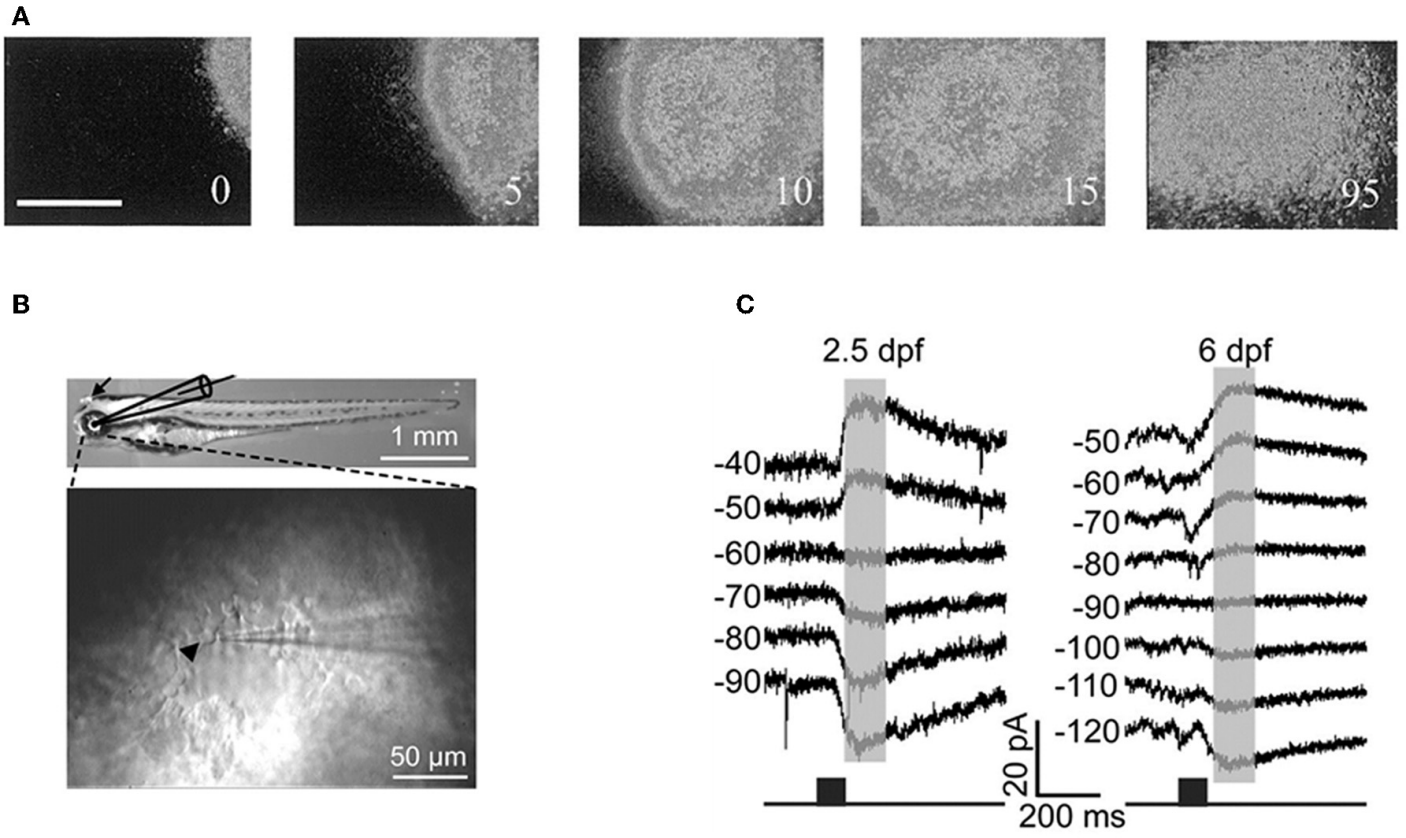
**GABA is excitatory in non-mammals retina both *in vivo* and *in vitro*. (A)** Time-lapse images of a GABA-induced wave in a S25 turtle retina. [Modified from Sernagor et al. (2003)]. **(B)** Diagram of *in vitro* whole-cell recording in intact zebrafish larvae. Top: bright-field image of whole-body morphology of a 3 dpf larva with removal of the lens (arrow). **(C)** GABA-induced currents recorded from two Retinal ganglion cells by using gramicidin perforated-patch whole-cell recording [left: 2.5 days post-fertilization (dpf); right: 6 dpf] at different membrane potentials (in mV). The currents were evoked by focal application of GABA (black squares) near RGC soma. Note the developmental shift in the reversal potential of GABA-induced currents. [**B** and **C** modified from Zhang et al. (2010)].

Cold-blooded vertebrates are far more resistant to anoxia and metabolic stress than mammals. Yet, in the developing turtle retina, GABA depolarizes ganglion cells in the embryo and becomes involved in controlling the dynamics of retinal waves at embryonic stage 25, one week before hatching (S26) (Sernagor et al., 2003) (Figure 8A). Interestingly, bath application of low doses of bicuculline at S25 completely blocks the occurrence of the waves, suggesting that GABA provides a baseline level of excitation that is necessary to trigger the waves. GABA gradually switches to become inhibitory by hatching time, as reflected by a change in wave dynamics: waves gradually slow down and shrink, eventually becoming stationary patches. Bath application of bicuculline at hatching reverts the activity to propagating waves, as in earlier embryonic networks. GABA keeps slowly shifting to its full inhibitory power during the first few weeks post-hatching, hence the process is much slower than in mammals.

More recently, Zhang and collaborators performed *in vivo* gramicidin perforated patch clamp recording in intact zebrafish larvae to characterize the developmental change of GABA actions in retinal ganglions cells (RGCs) (Zhang et al., 2010). They reported that zebrafish RGCs exhibited a depolarizing-hyperpolarizing (D-H) shift in GABA action at 2.5 days post-fertilization and D-H switch is delayed following down-regulation of KCC2 (Figures 8B,C). These observations are consistent with the developmental profile of KKC2 expression in zebrafish and with the neurogenic roles of depolarizing chloride revealed by global early over-expression of KCC2 (Reynolds et al., 2008). These observations further invalidate the suggestions of Bregestovski and Bernard.

## The issue of *In vivo* studies in rodents

**Figure 9:**
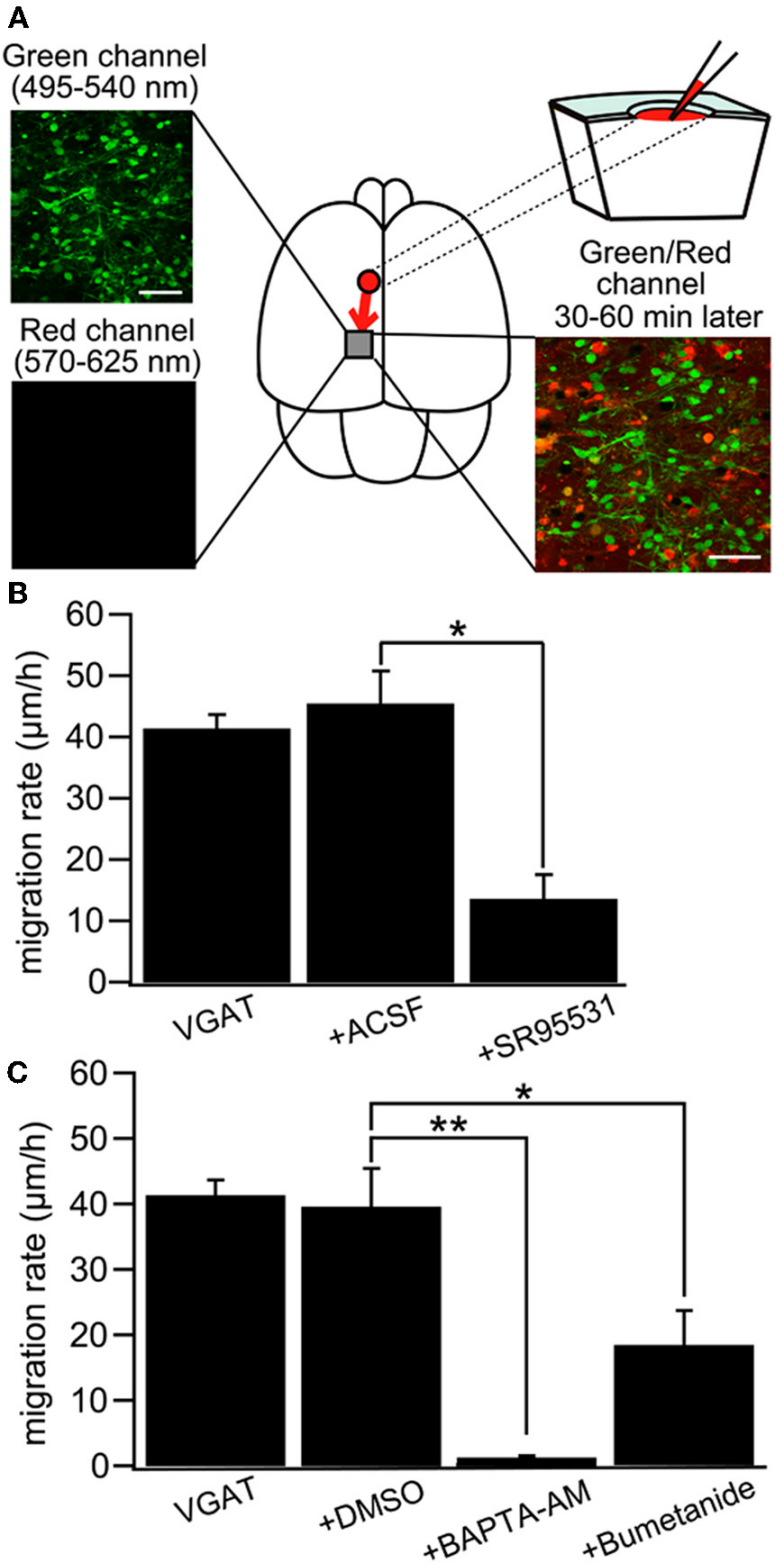
**GABA regulates tangential migration in the immature cortex *in vivo*. (A)** *Upper left:* Two-photon image of Venus positive cells immediately after drug application in a P0 VGAT-Venus mouse. Making a hole at 1–2 mm away from the observed area has no effect on the morphology of the cells within imaging area. Scale bar = 50 μm. *Lower left:* Two-photon image of sulforhodamine 101 (SR101) showing that the drug does not reach the imaging area immediately after injection. *Upper right:* Schematic drawing of the drug injection procedure; making a hole and injecting the drug conjugated with SR101. The drug diffuses into the imaging area after 30–60 minutes of injection. *Lower right:* Two-photon image of Venus-positive cells and SR101 showing that the pipette solution spread around the cells without injuring them. Scale bar = 50 μm. **(B)** Cell displacement during 1 h of migration for neurons in VGAT-Venus without drug application (VGAT), after injection of ACSF (+ACSF) and after injection of SR95531 (+SR95531). The selective GABA_A_R antagonist (SR95531, 1 mM, 1 μl) reduced the migration rate significantly (^*^p < 0.05) whereas ACSF (1 μl) had no effect. **(C)** DMSO (1 μl) had no effect on cell displacement. Chelating intracellular Ca^2+^ by BAPTA-AM (2 mM, 1 μl) conjugated with DMSO almost completely inhibited the migration (^**^p < 0.01) and blockade of NKCC by bumetanide (1 mM, 1 μl) significantly reduced the migration rate (^*^p < 0.05). [From Inada et al. (2011)].

Intuitively, *in vivo* studies are needed to confirm or reject the depolarizing/excitatory actions of GABA. Yet, things are more complex and *in vivo* studies particularly in rodent pups suffer from substantive severe potential handicaps. It is not possible to perform chronic recordings during the first postnatal days because of brain and bone growth. Many anaesthetic agents shift the polarity of GABA and augment inhibition rendering the interpretation of results obtained in these conditions difficult (Desfeux et al., 2010; Ando et al., 2011; McNally et al., 2011). Acute un-anaesthetized recordings generate even more complications both because of pain and ethical issues but also due to the release of adrenaline and other stress agents that may shift the polarity of GABA actions. Comparing *in vitro* experiments where E_GABA_ was determined in adequately controlled conditions to the excitation/inhibition produced by GABA agonists on extracellular recording of firing is of limited value since two different parameters are being measured. The difficulty of recordings from identified neuronal populations is yet another severe handicap. Yet, in spite of these limitations, several *in vivo* studies confirm the developmental shift. Tangential migration of GABA neurons is dependent on depolarizing GABA_A_ receptor activation and a reduction in intracellular Cl^−^ by KCC2 expression is sufficient for reducing interneuron motility by decreasing GABA depolarization (Bortone and Polleux, 2009). This study was carried out by using slice culture, but the results are quite compatible to that of a recent study with newborn mice *in vivo* that indicated NKCC1-dependent ambient GABA-mediated depolarization facilitates multidirectional tangential migration of GABAergic interneurons lacking KCC2 expression (Inada et al., 2011). In this model, applications of GABA antagonists, the NKCC1 antagonist bumetanide or the calcium chelator BAPTA delayed tangential migration (Figure 9) [also see (Wang and Kriegstein, 2008)]. Interestingly, radial migration also requires high intracellular Cl^−^ as applications of GABA receptor antagonists to slice cultures or subdural to neonatal rats accelerated radial migration (Heck et al., 2007; Manent and Represa, 2007).

## Conclusions

The observations of the Zilberters and colleagues have been repeatedly contradicted and suffer from severe drawbacks. Perhaps most importantly, there are intrinsic contradictions between the references they put forward to substantiate their hypothesis and the conclusions drawn from these studies.

In conclusion, the overwhelming convergence of observations made in a wide range of animal species and preparations confirm the developmental sequence of the polarity of GABA as a genuine fundamental feature of developmental neurobiology. This is another illustration of the developmental sequences that occur with all voltage and transmitter gated currents reflecting the major differences between the developing and adult brain: the developing brain is not a small adult brain. The most compelling evidence in favor of this sequence is simply that it is present. The alternative concept—that GABA exerts the same hyperpolarizing action across the entire developmental stage- is not even suggested by Zilberter and colleagues as they consider the excitatory GABA actions on embryonic neurons non refutable. Challenging the shift would be incongruent with the developmental sequences of all voltage and transmitter gated currents and with the well demonstrated trophic roles of GABA at an early stage that relies on excitatory GABA. GABAergic currents are like others long lasting because of a different subunit composition and directly involved in fundamental developmental mechanisms including cell division, migration and synapse and network formation. These elements cannot be refuted on the basis of damage and/or metabolism. Good healthy neurons like the patterns they generate are readily identified by trained scientists relying on criteria that remain as good today as they were decades ago. The available preparations offer a wide range of resources to better understand the roles of depolarizing GABA and the modulation of the timing in different systems, there is no need to alter their composition.

### Conflict of interest statement

The authors declare that the research was conducted in the absence of any commercial or financial relationships that could be construed as a potential conflict of interest.

## References

[B1] Achilles K. Okabe A. Ikeda M. Shimizu-Okabe C. Yamada J. Fukuda A. Luhmann H. J. Kilb W. (2007). Kinetic properties of Cl uptake mediated by Na+-dependent K+-2Cl cotransport in immature rat neocortical neurons. J. Neurosci. 27, 8616–8627 10.1523/JNEUROSCI.5041-06.2007 17687039 PMC6672936

[B2] Acton B. A. Mahadevan V. Mercado A. Uvarov P. Ding Y. Pressey J. Airaksinen M. S. Mount D. B. Woodin M. A. (2012). Hyperolparzing GABAergic transmission requires the KCC2 C-terminal ISO domain. J. Neurosci. 32, 8746–8751 10.1523/JNEUROSCI.6089-11.2012 22723714 PMC3395202

[B3] Akerman C. J. Cline H. T. (2006). Depolarizing GABAergic conductances regulate the balance of excitation to inhibition in the developing retinotectal circuit *in vivo*. J. Neurosci. 26, 5117–5130 10.1523/JNEUROSCI.0319-06.2006 16687503 PMC6674233

[B4] Andersen P. Dingledine R. Gjerstad L. Langmoen I. A. Laursen A. M. (1980). Two different responses of hippocampal pyramidal cells to application of gamma-amino butyric acid. J. Physiol. 305, 279–296 7441554 10.1113/jphysiol.1980.sp013363 PMC1282972

[B5] Ando Y. Hojo M. Kanaide M. Takada M. Sudo Y. Shiraishi S. Sumikawa K. Uezono Y. (2011). S(+)-ketamine suppresses desensitization of gamma-aminobutyric acid type B receptor-mediated signaling by inhibition of the interaction of gamma-aminobutyric acid type B receptors with G protein-coupled receptor kinase 4 or 5. Anesthesiology 114, 401–411 10.1097/ALN.0b013e318204e003 21245733

[B6] Balena T. Woodin M. A. (2008). Coincident pre- and postsynaptic activity downregulates NKCC1 to hyperpolarize E(Cl) during development. Eur. J. Neurosci. 27, 2402–2412 10.1111/j.1460-9568.2008.06194.x 18430034

[B7] Banke T. G. McBain C. J. (2006). GABAergic input onto CA3 hippocampal interneurons remains shunting throughout development. J. Neurosci. 26, 11720–11725 10.1523/JNEUROSCI.2887-06.2006 17093093 PMC6674795

[B8] Baram T. Z. Snead O. C. (1990). Bicuculline induced seizures in infant rats: ontogeny of behavioral and electrocortical phenomena. Brain Res. Dev. Brain Res. 57, 291–295 2073726 10.1016/0165-3806(90)90055-4 PMC3139479

[B9] Barkis W. B. Ford K. J. Feller M. B. (2010). Non-cell-autonomous factor induces the transition from excitatory to inhibitory GABA signaling in retina independent of activity. Proc. Natl. Acad. Sci. U.S.A. 107, 22302–22307 10.1073/pnas.1008775108 21135238 PMC3009811

[B10] Ben-Ari Y. (2001). Developing networks play a similar melody. Trends Neurosci. 24, 353–360 10.1016/S0166-2236(00)01813-0 11356508

[B11] Ben-Ari Y. (2002). Excitatory actions of gaba during development: the nature of the nurture. Nat. Rev. Neurosci. 3, 728–739 10.1038/nrn920 12209121

[B12] Ben-Ari Y. Cherubini E. Corradetti R. Gaiarsa J. L. (1989). Giant synaptic potentials in immature rat CA3 hippocampal neurones. J. Physiol. 416, 303–325 2575165 10.1113/jphysiol.1989.sp017762 PMC1189216

[B13] Ben-Ari Y. Gaiarsa J. L. Tyzio R. Khazipov R. (2007). GABA: a pioneer transmitter that excites immature neurons and generates primitive oscillations. Physiol. Rev. 87, 1215–1284 10.1152/physrev.00017.2006 17928584

[B14] Ben-Ari Y. Khazipov R. Leinekugel X. Caillard O. Gaiarsa J. L. (1997). GABAA, NMDA and AMPA receptors: a developmentally regulated ‘menage a trois’. Trends Neurosci. 20, 523–529 10.1016/S0166-2236(97)01147-8 9364667

[B15] Blaesse P. Airaksinen M. S. Rivera C. Kaila K. (2009). Cation-chloride cotransporters and neuronal function. Neuron 61, 820–838 10.1016/j.neuron.2009.03.003 19323993

[B16] Blaesse P. Guillemin I. Schindler J. Schweizer M. Delpire E. Khiroug L. Friauf E. Nothwang H. G. (2006). Oligomerization of KCC2 correlates with development of inhibitory neurotransmission. J. Neurosci. 26, 10407–10419 10.1523/JNEUROSCI.3257-06.2006 17035525 PMC6674702

[B17] Bolteus A. J. Bordey A. (2004). GABA release and uptake regulate neuronal precursor migration in the postnatal subventricular zone. J. Neurosci. 24, 7623–7631 10.1523/JNEUROSCI.1999-04.2004 15342728 PMC6729616

[B18] Bonifazi P. Goldin M. Picardo M. A. Jorquera I. Cattani A. Bianconi G. Represa A. Ben-Ari Y. Cossart R. (2009). GABAergic hub neurons orchestrate synchrony in developing hippocampal networks. Science 326, 1419–1424 10.1126/science.1175509 19965761

[B19] Bortone D. Polleux F. (2009). KCC2 expression promotes the termination of cortical interneuron migration in a voltage-sensitive calcium-dependent manner. Neuron 62, 53–71 10.1016/j.neuron.2009.01.034 19376067 PMC3314167

[B20] Bos R. Vinay L. (2012). Glucose is an adequate energy substrate for the depolarizing action of GABA and glycine in the neonatal rat spinal cord *in vitro*. J. Neurophysiol. 107, 3107–3115 10.1152/jn.00571.2011 22457452

[B21] Branchereau P. Morin D. Bonnot A. Ballion B. Chapron J. Viala D. (2000). Development of lumbar rhythmic networks: from embryonic to neonate locomotor-like patterns in the mouse. Brain Res. Bull. 53, 711–718 10.1016/S0361-9230(00)00403-2 11165805

[B22] Bregestovski P. Bernard C. (2012). Excitatory GABA: how a correct observation may turn out to be an experimental artifact. Front. Pharmacol. 3:65 10.3389/fphar.2012.00065 22529813 PMC3329772

[B23] Bregestovski P. Waseem T. Mukhtarov M. (2009). Genetically encoded optical sensors for monitoring of intracellular chloride and chloride-selective channel activity. Front. Mol. Neurosci. 2:15 10.3389/neuro.02.015.2009 20057911 PMC2802328

[B24] Caillard O. Ben-Ari Y. Gaiarsa J. L. (1999). Mechanisms of induction and expression of long-term depression at GABAergic synapses in the neonatal rat hippocampus. J. Neurosci. 19, 7568–7577 10460263 10.1523/JNEUROSCI.19-17-07568.1999 PMC6782510

[B25] Carleton A. Petreanu L. T. Lansford R. Alvarez-Buylla A. Lledo P. M. (2003). Becoming a new neuron in the adult olfactory bulb. Nat. Neurosci. 6, 507–518 10.1038/nn1048 12704391

[B26] Chen G. Trombley P. Q. van den Pol A. N. (1996). Excitatory actions of GABA in developing rat hypothalamic neurones. J. Physiol. 494(Pt 2), 451–464 8842004 10.1113/jphysiol.1996.sp021505 PMC1160647

[B27] Cherubini E. Ben-Ari Y. Krnjevic K. (1989). Anoxia produces smaller changes in synaptic transmission, membrane potential, and input resistance in immature rat hippocampus. J. Neurophysiol. 62, 882–895 2809708 10.1152/jn.1989.62.4.882

[B28] Chiang P. H. Wu P. Y. Kuo T. W. Liu Y. C. Chan C. F. Chien T. C. Cheng J. K. Huang Y. Y. Chiu C. D. Lien C. C. (2012). GABA is depolarizing in hippocampal dentate granule cells of the adolescent and adult rats. J. Neurosci. 32, 62–67 10.1523/JNEUROSCI.3393-11.2012 22219270 PMC6621339

[B29] Chub N. O'Donovan M. J. (2001). Post-episode depression of GABAergic transmission in spinal neurons of the chick embryo. J. Neurophysiol. 85, 2166–2176 11353031 10.1152/jn.2001.85.5.2166

[B30] Chudotvorova I. Ivanov A. Rama S. Hubner C. A. Pellegrino C. Ben-Ari Y. Medina I. (2005). Early expression of KCC2 in rat hippocampal cultures augments expression of functional GABA synapses. J. Physiol. 566, 671–679 10.1113/jphysiol.2005.089821 15961425 PMC1464776

[B31] Cohen I. Navarro V. Clemenceau S. Baulac M. Miles R. (2002). On the origin of interictal activity in human temporal lobe epilepsy *in vitro*. Science 298, 1418–1421 10.1126/science.1076510 12434059

[B32] Coull J. A. Beggs S. Boudreau D. Boivin D. Tsuda M. Inoue K. Gravel C. Salter M. W. De K. Y. (2005). BDNF from microglia causes the shift in neuronal anion gradient underlying neuropathic pain. Nature 438, 1017–1021 10.1038/nature04223 16355225

[B33] Cserep C. Szabadits E. Szonyi A. Watanabe M. Freund T. F. Nyiri G. (2012). NMDA receptors in GABAergic synapses during postnatal development. PLoS ONE 7:e37753 10.1371/journal.pone.0037753 22662211 PMC3360635

[B34] Delpy A. Allain A. E. Meyrand P. Branchereau P. (2008). NKCC1 cotransporter inactivation underlies embryonic development of chloride-mediated inhibition in mouse spinal motoneuron. J. Physiol. 586, 1059–1075 10.1113/jphysiol.2007.146993 18096599 PMC2375629

[B35] Desfeux A. El G. F. Jegou S. Legros H. Marret S. Laudenbach V. Gonzalez B. J. (2010). Dual effect of glutamate on GABAergic interneuron survival during cerebral cortex development in mice neonates. Cereb. Cortex 20, 1092–1108 10.1093/cercor/bhp181 19759125

[B36] Dzhala V. Valeeva G. Glykys J. Khazipov R. Staley K. (2012). Traumatic alterations in GABA signaling disrupt hippocampal network activity in the developing brain. J. Neurosci. 32, 4017–4031 10.1523/JNEUROSCI.5139-11.2012 22442068 PMC3333790

[B37] Dzhala V. I. Staley K. J. (2003). Excitatory actions of endogenously released GABA contribute to initiation of ictal epileptiform activity in the developing hippocampus. J. Neurosci. 23, 1840–1846 12629188 10.1523/JNEUROSCI.23-05-01840.2003 PMC6741948

[B38] Fischer K. F. Lukasiewicz P. D. Wong R. O. (1998). Age-dependent and cell class-specific modulation of retinal ganglion cell bursting activity by GABA. J. Neurosci. 18, 3767–3778 9570807 10.1523/JNEUROSCI.18-10-03767.1998 PMC6793131

[B39] Fiumelli H. Cancedda L. Poo M. M. (2005). Modulation of GABAergic transmission by activity via postsynaptic Ca2+-dependent regulation of KCC2 function. Neuron 48, 773–786 10.1016/j.neuron.2005.10.025 16337915

[B40] Frick A. Feldmeyer D. Helmstaedter M. Sakmann B. (2008). Monosynaptic connections between pairs of L5A pyramidal neurons in columns of juvenile rat somatosensory cortex. Cereb. Cortex 18, 397–406 10.1093/cercor/bhm074 17548800

[B41] Funk K. Woitecki A. Franjic-Wurtz C. Gensch T. Mohrlen F. Frings S. (2008). Modulation of chloride homeostasis by inflammatory mediators in dorsal root ganglion neurons. Mol. Pain 4, 32 10.1186/1744-8069-4-32 18700020 PMC2526990

[B42] Galanopoulou A. S. (2008a). Dissociated gender-specific effects of recurrent seizures on GABA signaling in CA1 pyramidal neurons: role of GABA(A) receptors. J. Neurosci. 28, 1557–1567 10.1523/JNEUROSCI.5180-07.2008 18272677 PMC6671546

[B43] Galanopoulou A. S. (2008b). Sexually dimorphic expression of KCC2 and GABA function. Epilepsy Res. 80, 99–113 10.1016/j.eplepsyres.2008.04.013 18524541 PMC2613346

[B44] Galanopoulou A. S. Kyrozis A. Claudio O. I. Stanton P. K. Moshe S. L. (2003). Sex-specific KCC2 expression and GABA(A) receptor function in rat substantia nigra. Exp. Neurol. 183, 628–637 10.1016/S0014-4886(03)00213-9 14552904

[B45] Ganguly K. Schinder A. F. Wong S. T. Poo M. (2001). GABA itself promotes the developmental switch of neuronal GABAergic responses from excitation to inhibition. Cell 105, 521–532 10.1016/S0092-8674(01)00341-5 11371348

[B46] Gao X. B. Chen G. van den Pol A. N. (1998). GABA-dependent firing of glutamate-evoked action potentials at AMPA/kainate receptors in developing hypothalamic neurons. J. Neurophysiol. 79, 716–726 9463435 10.1152/jn.1998.79.2.716

[B47] Gao X. B. van den Pol A. N. (2000). GABA release from mouse axonal growth cones. J. Physiol. 523(Pt 3), 629–637 10.1111/j.1469-7793.2000.t01-1-00629.x 10718743 PMC2269824

[B48] Gao X. B. van den Pol A. N. (2001). GABA, not glutamate, a primary transmitter driving action potentials in developing hypothalamic neurons. J. Neurophysiol. 85, 425–434 11152743 10.1152/jn.2001.85.1.425

[B49] Gascon E. Dayer A. G. Sauvain M. O. Potter G. Jenny B. De R. M. Zgraggen E. Demaurex N. Muller D. Kiss J. Z. (2006). GABA regulates dendritic growth by stabilizing lamellipodia in newly generated interneurons of the olfactory bulb. J. Neurosci. 26, 12956–12966 10.1523/JNEUROSCI.4508-06.2006 17167085 PMC6674946

[B50] Ge S. Goh E. L. Sailor K. A. Kitabatake Y. Ming G. L. Song H. (2006). GABA regulates synaptic integration of newly generated neurons in the adult brain. Nature 439, 589–593 10.1038/nature04404 16341203 PMC1420640

[B51] Gonzalez-Islas C. Chub N. Garcia-Bereguiain M. A. Wenner P. (2010). GABAergic synaptic scaling in embryonic motoneurons is mediated by a shift in the chloride reversal potential. J. Neurosci. 30, 13016–13020 10.1523/JNEUROSCI.1659-10.2010 20881119 PMC2950003

[B52] Gonzalez-Islas C. Chub N. Wenner P. (2009). NKCC1 and AE3 appear to accumulate chloride in embryonic motoneurons. J. Neurophysiol. 101, 507–518 10.1152/jn.90986.2008 19036864 PMC2657071

[B53] Gozlan H. Ben-Ari Y. (2003). Interneurons are the source and the targets of the first synapses formed in the rat developing hippocampal circuit. Cereb. Cortex 13, 684–692 12764045 10.1093/cercor/13.6.684

[B54] Gulledge A. T. Stuart G. J. (2003). Excitatory actions of GABA in the cortex. Neuron 37, 299–309 10.1016/S0896-6273(02)01146-7 12546824

[B55] Gulyas A. I. Sik A. Payne J. A. Kaila K. Freund T. F. (2001). The KCl cotransporter, KCC2, is highly expressed in the vicinity of excitatory synapses in the rat hippocampus. Eur. J. Neurosci. 13, 2205–2217 10.1046/j.0953-816x.2001.01600.x 11454023

[B56] Hanson M. G. Landmesser L. T. (2003). Characterization of the circuits that generate spontaneous episodes of activity in the early embryonic mouse spinal cord. J. Neurosci. 23, 587–600 12533619 10.1523/JNEUROSCI.23-02-00587.2003 PMC6741864

[B57] He S. Ma J. Liu N. Yu X. (2010). Early enriched environment promotes neonatal GABAergic neurotransmission and accelerates synapse maturation. J. Neurosci. 30, 7910–7916 10.1523/JNEUROSCI.6375-09.2010 20534839 PMC6632687

[B58] Heck N. Kilb W. Reiprich P. Kubota H. Furukawa T. Fukuda A. Luhmann H. J. (2007). GABA-A receptors regulate neocortical neuronal migration *in vitro* and *in vivo*. Cereb. Cortex 17, 138–148 10.1093/cercor/bhj135 16452638

[B59] Hennig M. H. Grady J. van C. J. Sernagor E. (2011). Age-dependent homeostatic plasticity of GABAergic signaling in developing retinal networks. J. Neurosci. 31, 12159–12164 10.1523/JNEUROSCI.3112-11.2011 21865458 PMC4868139

[B60] Hennou S. Khalilov I. Diabira D. Ben-Ari Y. Gozlan H. (2002). Early sequential formation of functional GABA(A) and glutamatergic synapses on CA1 interneurons of the rat foetal hippocampus. Eur. J. Neurosci. 16, 197–208 10.1046/j.1460-9568.2002.02073.x 12169102

[B61] Holmgren C. D. Mukhtarov M. Malkov A. E. Popova I. Y. Bregestovski P. Zilberter Y. (2010). Energy substrate availability as a determinant of neuronal resting potential, GABA signaling and spontaneous network activity in the neonatal cortex *in vitro*. J. Neurochem. 112, 900–912 10.1111/j.1471-4159.2009.06506.x 19943846

[B62] Huberfeld G. Wittner L. Clemenceau S. Baulac M. Kaila K. Miles R. Rivera C. (2007). Perturbed chloride homeostasis and GABAergic signaling in human temporal lobe epilepsy. J. Neurosci. 27, 9866–9873 10.1523/JNEUROSCI.2761-07.2007 17855601 PMC6672644

[B63] Inada H. Watanabe M. Uchida T. Ishibashi H. Wake H. Nemoto T. Yanagawa Y. Fukuda A. Nabekura J. (2011). GABA regulates the multidirectional tangential migration of GABAergic interneurons in living neonatal mice. PLoS ONE 6:e27048 10.1371/journal.pone.0027048 22180776 PMC3236753

[B64] Ivanov A. Mukhtarov M. Bregestovski P. Zilberter Y. (2011). Lactate effectively covers energy demands during neuronal network activity in neonatal hippocampal slices. Front. Neuroenergetics 3:2 10.3389/fnene.2011.00002 21602909 PMC3092068

[B65] Jean-Xavier C. Pflieger J. F. Liabeuf S. Vinay L. (2006). Inhibitory postsynaptic potentials in lumbar motoneurons remain depolarizing after neonatal spinal cord transection in the rat. J. Neurophysiol. 96, 2274–2281 10.1152/jn.00328.2006 16807348

[B66] Johnson J. Tian N. Caywood M. S. Reimer R. J. Edwards R. H. Copenhagen D. R. (2003). Vesicular neurotransmitter transporter expression in developing postnatal rodent retina: GABA and glycine precede glutamate. J. Neurosci. 23, 518–529 12533612 10.1523/JNEUROSCI.23-02-00518.2003 PMC6741860

[B67] Khakhalin A. S. Aizenman C. D. (2012). GABAergic transmission and chloride equilibrium potential are not modulated by pyruvate in the developing optic tectum of *Xenopus laevis* tadpoles. PLoS ONE 7:e34446 10.1371/journal.pone.0034446 22496804 PMC3319581

[B68] Khalilov I. Chazal G. Chudotvorova I. Pellegrino C. Corby S. Ferrand N. Gubkina O. Nardou R. Tyzio R. Yamamoto S. Jentsch T. J. Hubner C. A. Gaiarsa J. L. Ben-Ari Y. Medina I. (2011). Enhanced synaptic activity and epileptiform events in the embryonic KCC2 deficient hippocampus. Front. Cell Neurosci. 5:23 10.3389/fncel.2011.00023 22065950 PMC3206525

[B69] Khalilov I. Esclapez M. Medina I. Aggoun D. Lamsa K. Leinekugel X. Khazipov R. Ben-Ari Y. (1997). A novel *in vitro* preparation: the intact hippocampal formation. Neuron 19, 743–749 10.1016/S0896-6273(00)80956-3 9354321

[B70] Khazipov R. Esclapez M. Caillard O. Bernard C. Khalilov I. Tyzio R. Hirsch J. Dzhala V. Berger B. Ben-Ari Y. (2001). Early development of neuronal activity in the primate hippocampus in utero. J. Neurosci. 21, 9770–9781 11739585 10.1523/JNEUROSCI.21-24-09770.2001 PMC6763061

[B71] Khazipov R. Leinekugel X. Khalilov I. Gaiarsa J. L. Ben-Ari Y. (1997). Synchronization of GABAergic interneuronal network in CA3 subfield of neonatal rat hippocampal slices. J. Physiol. 498(Pt 3), 763–772 9051587 10.1113/jphysiol.1997.sp021900 PMC1159192

[B72] Khirug S. Huttu K. Ludwig A. Smirnov S. Voipio J. Rivera C. Kaila K. Khiroug L. (2005). Distinct properties of functional KCC2 expression in immature mouse hippocampal neurons in culture and in acute slices. Eur. J. Neurosci. 21, 899–904 10.1111/j.1460-9568.2005.03886.x 15787696

[B73] Khirug S. Yamada J. Afzalov R. Voipio J. Khiroug L. Kaila K. (2008). GABAergic depolarization of the axon initial segment in cortical principal neurons is caused by the Na-K-2Cl cotransporter NKCC1. J. Neurosci. 28, 4635–4639 10.1523/JNEUROSCI.0908-08.2008 18448640 PMC6670448

[B74] Kilb W. Ikeda M. Uchida K. Okabe A. Fukuda A. Luhmann H. J. (2002). Depolarizing glycine responses in Cajal-Retzius cells of neonatal rat cerebral cortex. Neuroscience 112, 299–307 10.1016/S0306-4522(02)00071-4 12044448

[B75] Kirmse K. Witte O. W. Holthoff K. (2010). GABA depolarizes immature neocortical neurons in the presence of the ketone body ß-hydroxybutyrate. J. Neurosci. 30, 16002–16007 10.1523/JNEUROSCI.2534-10.2010 21106838 PMC6633760

[B76] Kolbaev S. N. Achilles K. Luhmann H. J. Kilb W. (2011a). Effect of depolarizing GABA(A)-mediated membrane responses on excitability of Cajal-Retzius cells in the immature rat neocortex. J. Neurophysiol. 106, 2034–2044 10.1152/jn.00699.2010 21775719

[B77] Kolbaev S. N. Luhmann H. J. Kilb W. (2011b). Activity-dependent scaling of GABAergic excitation by dynamic Cl– changes in Cajal-Retzius cells. Pflugers Arch. 461, 557–565 10.1007/s00424-011-0935-4 21336585

[B78] Kraushaar U. Jonas P. (2000). Efficacy and stability of quantal GABA release at a hippocampal interneuron-principal neuron synapse. J. Neurosci. 20, 5594–5607 10908596 10.1523/JNEUROSCI.20-15-05594.2000 PMC6772523

[B79] Krnjevic K. Cherubini E. Ben-Ari Y. (1989). Anoxia on slow inward currents of immature hippocampal neurons. J. Neurophysiol. 62, 896–906 2553881 10.1152/jn.1989.62.4.896

[B80] Kudo N. Nishimaru H. Nakayama K. (2004). Developmental changes in rhythmic spinal neuronal activity in the rat fetus. Prog. Brain Res. 143, 49–55 14653150 10.1016/s0079-6123(03)43005-7

[B81] Kuner T. Augustine G. J. (2000). A genetically encoded ratiometric indicator for chloride: capturing chloride transients in cultured hippocampal neurons. Neuron 27, 447–459 10.1016/S0896-6273(00)00056-8 11055428

[B82] Lagostena L. Rosato-Siri M. D'Onofrio M. Brandi R. Arisi I. Capsoni S. Franzot J. Cattaneo A. Cherubini E. (2010). In the adult hippocampus, chronic nerve growth factor deprivation shifts GABAergic signaling from the hyperpolarizing to the depolarizing direction. J. Neurosci. 30, 885–893 10.1523/JNEUROSCI.3326-09.2010 20089897 PMC6633100

[B83] Landmesser L. T. O'Donovan M. J. (1984). Activation patterns of embryonic chick hind limb muscles recorded in ovo and in an isolated spinal cord preparation. J. Physiol. 347, 189–204 6707956 10.1113/jphysiol.1984.sp015061 PMC1199442

[B84] Le Magueresse C. Safiulina V. Changeux J. P. Cherubini E. (2006). Nicotinic modulation of network and synaptic transmission in the immature hippocampus investigated with genetically modified mice. J. Physiol. 576, 533–546 10.1113/jphysiol.2006.117572 16901939 PMC1890366

[B85] Lee H. H. Deeb T. Z. Walker J. A. Davies P. A. Moss S. J. (2011). NMDA receptor activity downregulates KCC2 resulting in depolarizing GABAA receptor-mediated currents. Nat. Neurosci. 14, 736–743 10.1038/nn.2806 21532577 PMC3102766

[B86] Lee H. H. Jurd R. Moss S. J. (2010). Tyrosine phosphorylation regulates the membrane trafficking of the potassium chloride co-transporter KCC2. Mol. Cell Neurosci. 45, 173–179 10.1016/j.mcn.2010.06.008 20600929 PMC3529177

[B87] Leinekugel X. Khalilov I. Ben-Ari Y. Khazipov R. (1998). Giant depolarizing potentials: the septal pole of the hippocampus paces the activity of the developing intact septohippocampal complex *in vitro*. J. Neurosci. 18, 6349–6357 9698326 10.1523/JNEUROSCI.18-16-06349.1998 PMC6793205

[B88] Leinekugel X. Khazipov R. Cannon R. Hirase H. Ben-Ari Y. Buzsaki G. (2002). Correlated bursts of activity in the neonatal hippocampus *in vivo*. Science 296, 2049–2052 10.1126/science.1071111 12065842

[B89] Leinekugel X. Medina I. Khalilov I. Ben-Ari Y. Khazipov R. (1997). Ca2+ oscillations mediated by the synergistic excitatory actions of GABA(A) and NMDA receptors in the neonatal hippocampus. Neuron 18, 243–255 10.1016/S0896-6273(00)80265-2 9052795

[B90] Leitch E. Coaker J. Young C. Mehta V. Sernagor E. (2005). GABA type-A activity controls its own developmental polarity switch in the maturing retina. J. Neurosci. 25, 4801–4805 10.1523/JNEUROSCI.0172-05.2005 15888655 PMC6724776

[B91] Li H. Khirug S. Cai C. Ludwig A. Blaesse P. Kolikova J. Afzalov R. Coleman S. K. Lauri S. Airaksinen M. S. Keinanen K. Khiroug L. Saarma M. Kaila K. Rivera C. (2007). KCC2 interacts with the dendritic cytoskeleton to promote spine development. Neuron 56, 1019–1033 10.1016/j.neuron.2007.10.039 18093524

[B92] Liu Z. Neff R. A. Berg D. K. (2006). Sequential interplay of nicotinic and GABAergic signaling guides neuronal development. Science 314, 1610–1613 10.1126/science.1134246 17158331

[B93] Lozada A. F. Wang X. Gounko N. V. Massey K. A. Duan J. Liu Z. Berg D. K. (2012). Glutamatergic synapse formation is promoted by alpha7-containing nicotinic acetylcholine receptors. J. Neurosci. 32, 7651–7661 10.1523/JNEUROSCI.6246-11.2012 22649244 PMC3370670

[B94] Manent J. B. Represa A. (2007). Neurotransmitters and brain maturation: early paracrine actions of GABA and glutamate modulate neuronal migration. Neuroscientist 13, 268–279 10.1177/1073858406298918 17519369

[B95] Mazzuca M. Minlebaev M. Shakirzyanova A. Tyzio R. Taccola G. Janackova S. Gataullina S. Ben-Ari Y. Giniatullin R. Khazipov R. (2011). Newborn analgesia mediated by oxytocin during delivery. Front. Cell Neurosci. 5:3 10.3389/fncel.2011.00003 21519396 PMC3080614

[B96] McLean H. A. Caillard O. Ben-Ari Y. Gaiarsa J. L. (1996). Bidirectional plasticity expressed by GABAergic synapses in the neonatal rat hippocampus. J. Physiol. 496(Pt 2), 471–477 8910230 10.1113/jphysiol.1996.sp021699 PMC1160891

[B97] McNally J. M. McCarley R. W. McKenna J. T. Yanagawa Y. Brown R. E. (2011). Complex receptor mediation of acute ketamine application on *in vitro* gamma oscillations in mouse prefrontal cortex: modeling gamma band oscillation abnormalities in schizophrenia. Neuroscience 199, 51–63 10.1016/j.neuroscience.2011.10.015 22027237 PMC3237956

[B98] Mercado A. Broumand V. Zandi-Nejad K. Enck A. H. Mount D. B. (2006). A C-terminal domain in KCC2 confers constitutive K+-Cl– cotransport. J. Biol. Chem. 281, 1016–1026 10.1074/jbc.M509972200 16291749

[B99] Mienville J. M. (1998). Persistent depolarizing action of GABA in rat Cajal-Retzius cells. J. Physiol. 512(Pt 3), 809–817 10.1111/j.1469-7793.1998.809bd.x 9769423 PMC2231241

[B100] Minlebaev M. Khazipov R. (2011). Antiepileptic effects of endogenous beta-hydroxybutyrate in suckling infant rats. Epilepsy Res. 95, 100–109 10.1016/j.eplepsyres.2011.03.003 21470827

[B101] Mohajerani M. H. Cherubini E. (2005). Spontaneous recurrent network activity in organotypic rat hippocampal slices. Eur. J. Neurosci. 22, 107–118 10.1111/j.1460-9568.2005.04198.x 16029200

[B102] Moyer J. R. Brown T. H. (2002). Patch clamp techniques applied to brain slices, in Patch-Clamp Analysis: Advanced Techniques, eds Walz W. Boulton A. A. Baker G. B. (Boston, USA: Harvard Press), 135–193

[B103] Mueller A. L. Taube J. S. Schwartzkroin P. A. (1984). Development of hyperpolarizing inhibitory postsynaptic potentials and hyperpolarizing response to gamma-aminobutyric acid in rabbit hippocampus studied *in vitro*. J. Neurosci. 4, 860–867 6707735 10.1523/JNEUROSCI.04-03-00860.1984 PMC6564832

[B104] Mukhtarov M. Ivanov A. Zilberter Y. Bregestovski P. (2011). Inhibition of spontaneous network activity in neonatal hippocampal slices by energy substrates is not correlated with intracellular acidification. J. Neurochem. 116, 316–321 10.1111/j.1471-4159.2010.07111.x 21083663

[B105] Nabekura J. Ueno T. Okabe A. Furuta A. Iwaki T. Shimizu-Okabe C. Fukuda A. Akaike N. (2002). Reduction of KCC2 expression and GABAA receptor-mediated excitation after *in vivo* axonal injury. J. Neurosci. 22, 4412–4417 12040048 10.1523/JNEUROSCI.22-11-04412.2002 PMC6758784

[B106] Nardou R. Yamamoto S. Chazal G. Bhar A. Ferrand N. Dulac O. Ben-Ari Y. Khalilov I. (2011). Neuronal chloride accumulation and excitatory GABA underlie aggravation of neonatal epileptiform activities by phenobarbital. Brain 134, 987–1002 10.1093/brain/awr041 21436113

[B107] O'Donovan M. J. (1999). The origin of spontaneous activity in developing networks of the vertebrate nervous system. Curr. Opin. Neurobiol. 9, 94–104 10.1016/S0959-4388(99)80012-9 10072366

[B108] Obata K. Oide M. Tanaka H. (1978). Excitatory and inhibitory actions of GABA and glycine on embryonic chick spinal neurons in culture. Brain Res. 144, 179–184 10.1016/0006-8993(78)90447-X 638760

[B109] Obrietan K. van den Pol A. N. (1995). GABA neurotransmission in the hypothalamus: developmental reversal from Ca2+ elevating to depressing. J. Neurosci. 15, 5065–5077 7623135 10.1523/JNEUROSCI.15-07-05065.1995 PMC6577894

[B110] Obrietan K. van den Pol A. N. (1996). Growth cone calcium elevation by GABA. J. Comp. Neurol. 372, 167–175 10.1002/(SICI)1096-9861(19960819)372:2<167::AID-CNE1>3.0.CO;2-1 8863123

[B111] Owens D. F. Boyce L. H. Davis M. B. Kriegstein A. R. (1996). Excitatory GABA responses in embryonic and neonatal cortical slices demonstrated by gramicidin perforated-patch recordings and calcium imaging. J. Neurosci. 16, 6414–6423 8815920 10.1523/JNEUROSCI.16-20-06414.1996 PMC6578913

[B112] Owens D. F. Kriegstein A. R. (2002). Is there more to GABA than synaptic inhibition? Nat. Rev. Neurosci. 3, 715–727 10.1038/nrn919 12209120

[B113] Payne J. A. Rivera C. Voipio J. Kaila K. (2003). Cation-chloride co-transporters in neuronal communication, development and trauma. Trends Neurosci. 26, 199–206 10.1016/S0166-2236(03)00068-7 12689771

[B114] Picardo M. A. Guigue P. Bonifazi P. Batista-Brito R. Allene C. Ribas A. Fishell G. Baude A. Cossart R. (2011). Pioneer GABA cells comprise a subpopulation of hub neurons in the developing hippocampus. Neuron 71, 695–709 10.1016/j.neuron.2011.06.018 21867885 PMC3163067

[B115] Platel J. C. Dave K. A. Bordey A. (2008). Control of neuroblast production and migration by converging GABA and glutamate signals in the postnatal forebrain. J. Physiol. 586, 3739–3743 10.1113/jphysiol.2008.155325 18467361 PMC2538924

[B116] Ren J. Greer J. J. (2003). Ontogeny of rhythmic motor patterns generated in the embryonic rat spinal cord. J. Neurophysiol. 89, 1187–1195 10.1152/jn.00539.2002 12626606

[B116a] Quilichini P. P. Le Van Quyen M. Ivanov A. Turner D. A. Carabalona A. Gozlan H. Esclapez M. Bernard C. (2012). Hub GABA neurons mediate gamma-frequency oscillations at ictal-like event onset in the immature hippocampus. Neuron 74, 57–64 10.1016/j.neuron.2012.01.026 22500630 PMC3328133

[B117] Reynolds A. Brustein E. Liao M. Mercado A. Babilonia E. Mount D. B. Drapeau P. (2008). Neurogenic role of the depolarizing chloride gradient revealed by global overexpression of KCC2 from the onset of development. J. Neurosci. 28, 1588–1597 10.1523/JNEUROSCI.3791-07.2008 18272680 PMC6671553

[B118] Rheims S. Minlebaev M. Ivanov A. Represa A. Khazipov R. Holmes G. L. Ben-Ari Y. Zilberter Y. (2008). Excitatory GABA in rodent developing neocortex *in vitro*. J. Neurophysiol. 100, 609–619 10.1152/jn.90402.2008 18497364

[B119] Rivera C. Voipio J. Payne J. A. Ruusuvuori E. Lahtinen H. Lamsa K. Pirvola U. Saarma M. Kaila K. (1999). The K+/Cl– co-transporter KCC2 renders GABA hyperpolarizing during neuronal maturation. Nature 397, 251–255 10.1038/16697 9930699

[B120] Ruusuvuori E. Kirilkin I. Pandya N. Kaila K. (2010). Spontaneous network events driven by depolarizing GABA action in neonatal hippocampal slices are not attributable to deficient mitochondrial energy metabolism. J. Neurosci. 30, 15638–15642 10.1523/JNEUROSCI.3355-10.2010 21084619 PMC6633692

[B121] Sauer J. F. Struber M. Bartos M. (2012). Interneurons provide circuit-specific depolarization and hyperpolarization. J. Neurosci. 32, 4224–4229 10.1523/JNEUROSCI.5702-11.2012 22442084 PMC6621207

[B122] Sedlacek J. (1985). Bicuculline activation of embryonic spontaneous motility. Physiol. Bohemoslov. 34, 33–39 3158011

[B123] Sernagor E. Young C. Eglen S. J. (2003). Developmental modulation of retinal wave dynamics: shedding light on the GABA saga. J. Neurosci. 23, 7621–7629 12930801 10.1523/JNEUROSCI.23-20-07621.2003 PMC6740765

[B124] Sipila S. T. Schuchmann S. Voipio J. Yamada J. Kaila K. (2006). The cation-chloride cotransporter NKCC1 promotes sharp waves in the neonatal rat hippocampus. J. Physiol. 573, 765–773 10.1113/jphysiol.2006.107086 16644806 PMC1779742

[B125] Staiger J. F. Flagmeyer I. Schubert D. Zilles K. Kotter R. Luhmann H. J. (2004). Functional diversity of layer IV spiny neurons in rat somatosensory cortex: quantitative morphology of electrophysiologically characterized and biocytin labeled cells. Cereb. Cortex 14, 690–701 10.1093/cercor/bhh029 15054049

[B126] Staley K. J. Mody I. (1992). Shunting of excitatory input to dentate gyrus granule cells by a depolarizing GABAA receptor-mediated postsynaptic conductance. J. Neurophysiol. 68, 197–212 1381418 10.1152/jn.1992.68.1.197

[B127] Stil A. Jean-Xavier C. Liabeuf S. Brocard C. Delpire E. Vinay L. Viemari J. C. (2011). Contribution of the potassium-chloride co-transporter KCC2 to the modulation of lumbar spinal networks in mice. Eur. J. Neurosci. 33, 1212–1222 10.1111/j.1460-9568.2010.07592.x 21255132

[B128] Stil A. Liabeuf S. Jean-Xavier C. Brocard C. Viemari J. C. Vinay L. (2009). Developmental up-regulation of the potassium-chloride cotransporter type 2 in the rat lumbar spinal cord. Neuroscience 164, 809–821 10.1016/j.neuroscience.2009.08.035 19699273

[B129] Succol F. Fiumelli H. Benfenati F. Cancedda L. Barberis A. (2012). Intracellular chloride concentration influences the GABAA receptor subunit composition. Nat. Commun. 3, 738 10.1038/ncomms1744 22415829 PMC3316884

[B130] Sun Y. G. Wu C. S. Renger J. J. Uebele V. N. Lu H. C. Beierlein M. (2012). GABAergic synaptic transmission triggers action potentials in thalamic reticular nucleus neurons. J. Neurosci. 32, 7782–7790 10.1523/JNEUROSCI.0839-12.2012 22674255 PMC3376355

[B131] Sung K. W. Kirby M. McDonald M. P. Lovinger D. M. Delpire E. (2000). Abnormal GABAA receptor-mediated currents in dorsal root ganglion neurons isolated from Na-K-2Cl cotransporter null mice. J. Neurosci. 20, 7531–7538 11027211 10.1523/JNEUROSCI.20-20-07531.2000 PMC6772871

[B132] Takano H. McCartney M. Ortinski P. I. Yue C. Putt M. E. Coulter D. A. (2012). Deterministic and stochastic neuronal contributions to distinct synchronous CA3 network bursts. J. Neurosci. 32, 4743–4754 10.1523/JNEUROSCI.4277-11.2012 22492030 PMC3328771

[B133] Taylor M. Young S. Z. Wu S. Bordey A. (2011). Dampening GABA-A excitation in subventricular zone neural progenitor cell decreases neonatal neurogenesis. Soc. Neurosci. 31–16 Ref Type: Abstract.20379900

[B134] Tornberg J. Voikar V. Savilahti H. Rauvala H. Airaksinen M. S. (2005). Behavioural phenotypes of hypomorphic KCC2-deficient mice. Eur. J. Neurosci. 21, 1327–1337 10.1111/j.1460-9568.2005.03959.x 15813942

[B135] Toyoda H. Ohno K. Yamada J. Ikeda M. Okabe A. Sato K. Hashimoto K. Fukuda A. (2003). Induction of NMDA and GABAA receptor-mediated Ca2+ oscillations with KCC2 mRNA downregulation in injured facial motoneurons. J. Neurophysiol. 89, 1353–1362 10.1152/jn.00721.2002 12612004

[B136] Tyzio R. Allene C. Nardou R. Picardo M. A. Yamamoto S. Sivakumaran S. Caiati M. D. Rheims S. Minlebaev M. Milh M. Ferre P. Khazipov R. Romette J. L. Lorquin J. Cossart R. Khalilov I. Nehlig A. Cherubini E. Ben-Ari Y. (2011). Depolarizing actions of GABA in immature neurons depend neither on ketone bodies nor on pyruvate. J. Neurosci. 31, 34–45 10.1523/JNEUROSCI.3314-10.2011 21209187 PMC6622726

[B137] Tyzio R. Cossart R. Khalilov I. Minlebaev M. Hubner C. A. Represa A. Ben-Ari Y. Khazipov R. (2006). Maternal oxytocin triggers a transient inhibitory switch in GABA signaling in the fetal brain during delivery. Science 314, 1788–1792 10.1126/science.1133212 17170309

[B138] Tyzio R. Minlebaev M. Rheims S. Ivanov A. Jorquera I. Holmes G. L. Zilberter Y. Ben-Ari Y. Khazipov R. (2008). Postnatal changes in somatic gamma-aminobutyric acid signalling in the rat hippocampus. Eur. J. Neurosci. 27, 2515–2528 10.1111/j.1460-9568.2008.06234.x 18547241

[B139] Tyzio R. Represa A. Jorquera I. Ben-Ari Y. Gozlan H. Aniksztejn L. (1999). The establishment of GABAergic and glutamatergic synapses on CA1 pyramidal neurons is sequential and correlates with the development of the apical dendrite. J. Neurosci. 19, 10372–10382 10575034 10.1523/JNEUROSCI.19-23-10372.1999 PMC6782402

[B140] Uvarov P. Ludwig A. Markkanen M. Soni S. Hubner C. A. Rivera C. Airaksinen M. S. (2009). Coexpression and heteromerization of two neuronal K-Cl cotransporter isoforms in neonatal brain. J. Biol. Chem. 284, 13696–13704 10.1074/jbc.M807366200 19307176 PMC2679471

[B141] Valeeva G. Abdullin A. Tyzio R. Skorinkin A. Nikolski E. Ben-Ari Y. Khazipov R. (2010). Temporal coding at the immature depolarizing GABAergic synapse. Front. Cell Neurosci. 4:17 10.3389/fncel.2010.00017 20725525 PMC2914581

[B142] Valeeva G. Valiullina F. Khazipov R. (2012). Excitatory actions of GABA in the intact neonatal rodent hippocampus *in vitro*, in FENS Meeting, (Barcelona, Spain), abtract 1116. 10.3389/fncel.2013.00020 PMC3587803 23467988

[B143] van den Pol A. N. (1997). GABA immunoreactivity in hypothalamic neurons and growth cones in early development *in vitro* before synapse formation. J. Comp. Neurol. 383, 178–188 10.1002/(SICI)1096-9861(19970630)383:2<178::AID-CNE5>3.0.CO;2-Y 9182847

[B144] van den Pol A. N. Gao X. B. Patrylo P. R. Ghosh P. K. Obrietan K. (1998). Glutamate inhibits GABA excitatory activity in developing neurons. J. Neurosci. 18, 10749–10761 9852609 10.1523/JNEUROSCI.18-24-10749.1998 PMC6793361

[B145] van den Pol A. N. Obrietan K. Chen G. (1996). Excitatory actions of GABA after neuronal trauma. J. Neurosci. 16, 4283–4292 8753889 10.1523/JNEUROSCI.16-13-04283.1996 PMC6578987

[B146] Voigt T. Opitz T. de Lima A. D. (2001). Synchronous oscillatory activity in immature cortical network is driven by GABAergic preplate neurons. J. Neurosci. 21, 8895–8905 11698601 10.1523/JNEUROSCI.21-22-08895.2001 PMC6762259

[B147] Waddell J. Kim J. Alger B. E. McCarthy M. M. (2011). The depolarizing action of GABA in cultured hippocampal neurons is not due to the absence of ketone bodies. PLoS ONE 6:e23020 10.1371/journal.pone.0023020 21886776 PMC3158756

[B148] Wagner S. Castel M. Gainer H. Yarom Y. (1997). GABA in the mammalian suprachiasmatic nucleus and its role in diurnal rhythmicity. Nature 387, 598–603 10.1038/42468 9177347

[B149] Wang C. Ohno K. Furukawa T. Ueki T. Ikeda M. Fukuda A. Sato K. (2005). Differential expression of KCC2 accounts for the differential GABA responses between relay and intrinsic neurons in the early postnatal rat olfactory bulb. Eur. J. Neurosci. 21, 1449–1455 10.1111/j.1460-9568.2005.03975.x 15813956

[B150] Wang D. D. Kriegstein A. R. (2008). GABA regulates excitatory synapse formation in the neocortex via NMDA receptor activation. J. Neurosci. 28, 5547–5558 10.1523/JNEUROSCI.5599-07.2008 18495889 PMC2684685

[B151] Watanabe M. Wake H. Moorhouse A. J. Nabekura J. (2009). Clustering of neuronal K+-Cl– cotransporters in lipid rafts by tyrosine phosphorylation. J. Biol. Chem. 284, 27980–27988 10.1074/jbc.M109.043620 19679663 PMC2788850

[B152] Wilhelm J. C. Wenner P. (2008). GABAA transmission is a critical step in the process of triggering homeostatic increases in quantal amplitude. Proc. Natl. Acad. Sci. U.S.A. 105, 11412–11417 10.1073/pnas.0806037105 18678897 PMC2516260

[B153] Woo N. S. Lu J. England R. McClellan R. Dufour S. Mount D. B. Deutch A. Y. Lovinger D. M. Delpire E. (2002). Hyperexcitability and epilepsy associated with disruption of the mouse neuronal-specific K-Cl cotransporter gene. Hippocampus 12, 258–268 10.1002/hipo.10014 12000122

[B154] Yamada J. Okabe A. Toyoda H. Kilb W. Luhmann H. J. Fukuda A. (2004). Cl– uptake promoting depolarizing GABA actions in immature rat neocortical neurones is mediated by NKCC1. J. Physiol. 557, 829–841 10.1113/jphysiol.2004.062471 15090604 PMC1665166

[B155] Yang H. Shew W. L. Roy R. Plenz D. (2012). Maximal variability of phase synchrony in cortical networks with neuronal avalanches. J. Neurosci. 32, 1061–1072 10.1523/JNEUROSCI.2771-11.2012 22262904 PMC3319677

[B156] Yang J. W. Hanganu-Opatz I. L. Sun J. J. Luhmann H. J. (2009). Three patterns of oscillatory activity differentially synchronize developing neocortical networks *in vivo*. J. Neurosci. 29, 9011–9025 10.1523/JNEUROSCI.5646-08.2009 19605639 PMC6665441

[B157] Yeo M. Berglund K. Augustine G. Liedtke W. (2009). Novel repression of Kcc2 transcription by REST-RE-1 controls developmental switch in neuronal chloride. J. Neurosci. 29, 14652–14662 10.1523/JNEUROSCI.2934-09.2009 19923298 PMC2833346

[B158] Young S. Z. Platel J. C. Nielsen J. V. Jensen N. A. Bordey A. (2010). GABA(A) Increases calcium in subventricular zone astrocyte-like cells through L- and T-type voltage-gated calcium channels. Front. Cell Neurosci. 4:8 10.3389/fncel.2010.00008 20422045 PMC2857959

[B159] Yvert B. Branchereau P. Meyrand P. (2004). Multiple spontaneous rhythmic activity patterns generated by the embryonic mouse spinal cord occur within a specific developmental time window. J. Neurophysiol. 91, 2101–2109 10.1152/jn.01095.2003 14724265

[B160] Yvert B. Mazzocco C. Joucla S. Langla A. Meyrand P. (2011). Artificial CSF motion ensures rhythmic activity in the developing CNS *ex vivo*: a mechanical source of rhythmogenesis? J. Neurosci. 31, 8832–8840 10.1523/JNEUROSCI.1354-11.2011 21677167 PMC6622937

[B161] Zhang L. L. Pathak H. R. Coulter D. A. Freed M. A. Vardi N. (2006). Shift of intracellular chloride concentration in ganglion and amacrine cells of developing mouse retina. J. Neurophysiol. 95, 2404–2416 10.1152/jn.00578.2005 16371454

[B162] Zhang R. W. Wei H. P. Xia Y. M. Du J. L. (2010). Development of light response and GABAergic excitation-to-inhibition switch in zebrafish retinal ganglion cells. J. Physiol. 588, 2557–2569 10.1113/jphysiol.2010.187088 20498234 PMC2916988

[B163] Zilberter Y. Zilberter T. Bregestovski P. (2010). Neuronal activity *in vitro* and the *in vivo* reality: the role of energy homeostasis. Trends Pharmacol. Sci. 31, 394–401 10.1016/j.tips.2010.06.005 20633934

